# Transcription is a major driving force for plastid genome instability in Arabidopsis

**DOI:** 10.1371/journal.pone.0214552

**Published:** 2019-04-03

**Authors:** Juliana Andrea Pérez Di Giorgio, Étienne Lepage, Samuel Tremblay-Belzile, Sébastien Truche, Audrey Loubert-Hudon, Normand Brisson

**Affiliations:** Department of Biochemistry and Molecular Medicine, Université de Montréal, Montréal, Québec, Canada; Agriculture and Agri-Food Canada, CANADA

## Abstract

Though it is an essential process, transcription can be a source of genomic instability. For instance, it may generate RNA:DNA hybrids as the nascent transcript hybridizes with the complementary DNA template. These hybrids, called R-loops, act as a major cause of replication fork stalling and DNA breaks. In this study, we show that lowering transcription and R-loop levels in plastids of *Arabidopsis thaliana* reduces DNA rearrangements and mitigates plastid genome instability phenotypes. This effect can be observed on a genome-wide scale, as the loss of the plastid sigma transcription factor SIG6 prevents DNA rearrangements by favoring conservative repair in the presence of ciprofloxacin-induced DNA damage or in the absence of plastid genome maintenance actors such as WHY1/WHY3, RECA1 and POLIB. Additionally, resolving R-loops by the expression of a plastid-targeted exogenous RNAse H1 produces similar results. We also show that highly-transcribed genes are more susceptible to DNA rearrangements, as increased transcription of the psbD operon by SIG5 correlates with more locus-specific rearrangements. The effect of transcription is not specific to Sigma factors, as decreased global transcription levels by mutation of heat-stress-induced factor *HSP21*, mutation of nuclear-encoded polymerase *RPOTp*, or treatment with transcription-inhibitor rifampicin all prevent the formation of plastid genome rearrangements, especially under induced DNA damage conditions.

## Introduction

Plastids form a large family of cellular organelles in plants and algae that includes chloroplasts, which are responsible for photosynthesis. Plastids of vascular plants originated from an ancestral cyanobacterial endosymbiont, and therefore possess their own genome [1]. The present-day genome of the plant Arabidopsis harbors only 133 genes coding for 37 tRNAs, 8 rRNAs and 88 proteins that are involved in plastid gene expression, photosynthesis, biosynthesis of fatty acids, pigments and amino acids, or with yet unknown functions [2]. Plastids contain multiple copies of the plastid chromosome, folded together with proteins and RNA into nucleoids [3].

Despite their small genomes (~0.15 Mbp in land plant plastids versus 3 Mbp in cyanobacteria), plastids possess complex hybrid gene expression systems composed of both prokaryotic and eukaryotic components. Plastid genes are transcribed into polycistronic RNAs that are subsequently processed into complex mRNA isoforms [4]. In Arabidopsis, plastid transcription is performed by at least three different RNA polymerases (RNAP): two phage-type nucleus-encoded polymerases (NEP: RPOTp and RPOTmp) and one bacterial-type plastid-encoded polymerase (PEP), composed of four basic core subunits encoded by the plastid genes rpoA, rpoB, rpoC1, and rpoC2 [5].

NEP and PEP recognize different types of promoters. While PEP promoters closely resemble bacterial σ^70^-type promoters and comprise a −35 and a −10 (TATA) box, most NEP promoters have a core sequence motif (YRTA) that is similar to the consensus sequence of promoters in plant mitochondrial genomes. Transcription of highly expressed photosynthesis-related genes (such as psbA, psbD, and rbcL) depends largely on PEP (class I), whereas a few house-keeping genes (such as rpoB, rpoC1, rpoC2, accD, and ycf2) are transcribed by NEPs (class III). However, most plastid genes contain promoter elements that are recognized by both polymerases (class II) [6].

NEP is more active in the youngest, non-green tissues early in chloroplast development and plays an especially important role in the transcription of PEP core subunits and genes for the translational machinery. Subsequently, NEP activity decreases and PEP dominates in the transcription of most mRNA and tRNA genes and plays an important role in rRNA synthesis [4]. PEP represents the major transcription machinery in mature chloroplasts and > 80% of all primary plastid transcripts appear to be transcribed by PEP [6].

The PEP requires additional nucleus-encoded sigma factors for promoter recognition and transcription initiation as well as further factors, which associate to the polymerase core during chloroplast development [7]. The six sigma factors (SIG1-6) identified in Arabidopsis have overlapping as well as specific functions for recognizing a specific set of promoters during plastid development [4,8,9]. Noteworthy, *SIG6* mutant plants display a pale-green chlorophyll-deficient, growth-retarded phenotype during the early stages of development, demonstrating its importance for chloroplast biogenesis [10,11]. Transcriptome analysis of 4-d-old *sig6* seedlings showed reduced transcript levels of most photosynthetic genes [10]. Additionally, SIG6 has a persistent role in the transcription of certain plastid operons (atpB/E, ndhC/psbG/ndhJ) [11,12]. This suggests a dual role of SIG6 consisting in both an early global and a long-term gene-specific activity.

In spite of the genotoxic stress and DNA damage applied by UV radiation from sunlight and reactive oxygen species (ROS) from photosynthesis [13], plastid genomes are surprisingly more stable than the nuclear genome at the nucleotide level [14,15]. Proteins involved in plastid DNA (ptDNA) stability include Whirly proteins WHY1 and WHY3, recombinase RECA1 and type-I polymerase POLIB. Whirly proteins suppress error-prone microhomology-mediated recombination (MHMR) via nonspecific binding to single-stranded DNA (ssDNA) [16–18]. In Arabidopsis, the double mutant *why1why3* presents high levels of ptDNA rearrangements mediated by MHMR, causing the development of white/yellow variegated leaves in about 5% of the plant population [16,18]. RECA1 maintains plastid genome stability through its central role in homologous recombination repair, especially in replication fork reversal [18,19]. POLIB also plays a role in ptDNA repair, and the *polIb* mutation in Arabidopsis causes replication stress at early developmental stages and increases the amount of DSBs upon genotoxic stress treatment [20].

Accumulating evidence indicates that transcription-replication conflicts are an important natural source of genome instability (reviewed in [21]). As replication and transcription compete for the same DNA template, collisions between both complexes, occurring either in co-directional or head-on orientation, can cause replication fork blocking and collapse, generating DSBs and genome instability [22]. In the last few years, transcription-associated R-loops have emerged as a major source of DNA-break-mediated genome instability (reviewed in [23,24]). R-loops are stable, three-stranded nucleic acid structures, composed of an extended RNA:DNA hybrid formed by reinvasion of the nascent RNA molecule into the template DNA strand and the displaced non-template DNA strand as a loop of ssDNA. R-loops constitute a unique threat because they can potentially obstruct replisome progression, which can result in replication fork collapse or breakage [25].

To preserve genome integrity, cells use various mechanisms to prevent replication-transcription collisions from occurring and to resolve R-loop conflicts once they have occurred [22]. For example, helicases and topoisomerases help to relieve topological stress generated between converging replication and transcription complexes, while RNA processing factors prevent the RNA transcript from interacting with the DNA template. In the event that rehybridization does occur, RNA:DNA helicases can unwind these structures, and RNase H can digest the RNA portion of an RNA:DNA hybrid. The Arabidopsis genome encodes three proteins with RNase H domains, two of the archaeal H1 type with predicted localizations to mitochondria (At5g51080) and plastids (At1g24090, named RNH1C), respectively, and one with weak similarities to the H2-type predicted to be localized to plastids (At5g61090) [26]. Most recently, Yang et al. (2017) showed that RNH1C maintains R-loop homeostasis and genome integrity in Arabidopsis plastids, suggesting a role of plastid transcription on ptDNA instability [27].

Although studies have shown that transcription accelerates the endogenous rate of DNA damage in bacteria, yeast, and mammals (reviewed in [24]), still little is known about its effect on plastids, which are of great interest as per their unique hybrid transcription system. In this study, we used various experimental conditions and numerous mutant lines, known to be defective in plastid transcription, to evaluate the impact of this process on plastid genome stability. Our results suggest that plastid transcription can threaten plastid genome stability by inducing R-loops and preventing conservative DNA repair and thus, needs to be tightly controlled.

## Materials and methods

### Plant material, cloning, transformation and growth conditions

*Arabidopsis thaliana* (ecotype Columbia-0) wild type (WT), T-DNA insertional mutant lines: *sig1-1* (SALK_088796) and *sig1-2* (SALK_147985) [28,29], *sig2-1* (SALK_045706) [29–31], *sig3-2* (SALK_009166) and *sig3-4* (SALK_081321) [29,32], *sig4-1* (SALK_027838) and *sig4-3* (SALK_078760) [29], *sig5-1* (SALK_049021) [33] and *sig5-2* (SALK_141383) [29,33], *sig6-1* (SAIL_645_F03) and *sig6-2* (SAIL_893_C09) [10,29,31], and the line *hsp21* (CS85472) mutated by ethyl methanesulfonate [34] were obtained from the ABRC (http://abrc.osu.edu/). Double mutant *why1why3* and triple mutants *why1why3polIb* and *why1why3reca1* have been previously described [16,17,20,35]. Triple mutant *why1why3sig6-1* and quadruple mutants *why1why3polIbsig6-1* and *why1why3reca1sig6-1* were obtained from a genetic cross between the above mentioned mutants and *sig6-1*. All mutant genotypes were confirmed by PCR and primer sequences are listed in S1 Table. Confirmed homozygous mutant plants were used in this study.

Arabidopsis transgenic line 347, which expresses a plastid-targeted endonuclease I-CREII from *Chlamydomonas reinhardtii* upon β-estradiol induction, was kindly provided by Dr. David L. Herrin (University of Texas, Austin). This line was used to cause a DSB at the psbA plastid gene intron according to the author’s protocol [36].

Arabidopsis transgenic lines expressing a plastid-targeted RNAse H1 from *Escherichia coli* upon β-estradiol induction were developed. Briefly, the coding sequence of *E*. *coli* RNAse H1 (PCN061) fused to the N-terminal (amino acids 1–59) transit peptide of the rbcS1 gene [37] was cloned into the pGPTVII binary plasmid harboring the inducible expression cassette: _P_SUPERR:XVE: _P_LexA+min.35S:MCS:StrepII:NosT [38] using BamHI and XhoI restriction enzymes. Plasmid constructs were checked by DNA sequencing and transformed into *Agrobacterium tumefaciens* strain GV3101, to allow transformation of *A*. *thaliana* Col-0 plants by the floral-dip method [39]. For selection of transgenic lines, seeds were sown on plates containing Murashige and Skoog (MS) basal media (Sigma-Aldrich) supplemented with 1% Sucrose, 0.8% agar and 25 mg/mL ammonium glufosinate. Four independent RNAse H1 inducible lines (named RNAse H1_A, B and C) and a control line transformed with the empty plasmid (named Ctl) were selected upon DNA sequencing and RT-PCR expression assays. All transgenic lines were confirmed by PCR and primer sequences are listed in S1 Table. Confirmed homozygous transgenic plants were used in this study.

Sterilized seeds were either sown on soil or placed on petri dishes containing 0.5X MS basal media supplemented with 1% Sucrose and 0.8% agar, with or without ciprofloxacin (0.50 μM, Sigma-Aldrich), rifampicin (100 μM, BioShop), and/or β-estradiol (10 or 50 μM, Sigma-Aldrich) as indicated. After 3 days of stratification at 4°C, seeds were placed under light (100 mmol m^–2^ s^–1^) at 22°C on a 16-h-day/8-h-dark cycle. *hsp21* mutant lines were grown as previously reported [34].

### PCR detection of DNA rearrangements

Plastid DNA rearrangements were detected as previously described [17]. Whole plants grown on MS media were harvested at 14 days after germination (five to six true leaves) by pulling the plants (including the roots) carefully from the agar and instantly frozen in liquid nitrogen. Plants were pooled to make 75-mg samples. DNA was isolated from plants using the cetyl trimethyl-ammonium bromide (CTAB) DNA extraction protocol [40] plus RNAse A treatment (Fermentas). PCR was conducted using the Taq polymerase (Genscript) according to the manufacturer’s instructions. DNA rearrangement events were detected using both outward- and inward-facing PCR primers spaced by 5 to 20 kb. Eight PCR primer pairs scattered in the plastid genome were used (S1 Table and S1 Fig). Low cycle amplifications of the atpB and ycf2 plastid genes were used as loading controls. For each PCR, the number of ptDNA rearrangements was estimated by quantifying the intensities of bands visualized on the same gel using the ChemiDoc^TM^ MP System (Bio-Rad).

### Illumina DNA sequencing

Total DNA was isolated from ~400 mg pools of leaves from 14-d-old plants grown on soil using the CTAB DNA extraction protocol [40] plus RNAse A treatment (Fermentas). Libraries were prepared from the DNA samples using the NxSeq AmpFREE Low DNA Library kit (LuciGen, Cat. #14000–2) as per the manufacturer’s protocol. This includes SPRI bead cleanup and size selection steps for a final median insert size of ~320 bp. Sequencing was performed on the Illumina HiSeq X PE150 (paired-end, 150bp). Library preparation and sequencing were done at Génome Québec (McGill University, Montréal, Qc, Canada). All datasets were made available on NCBI SRA (Project number: PRJNA482863).

### DNA-seq analysis of rearranged reads and plastid coverage

Sequences of reads spanning potential junctions were obtained following a Galaxy workflow and aligned to the organelle and nuclear genomes (GenBank: AP000423.1 for plastid, NC_037304.1 for mitochondria, and TAIR10 for nuclear genome) using BLAST+ as previously described [18]. Rearrangements with an overlap (subtraction of the total read length from the sum of the lengths of both alignments) of at least 5 bases were considered to have occurred through the use of microhomology (+MH), while the rest were assigned to “no microhomology” (-MH). Plastid sequencing coverage and quantitative PCR analysis of ptDNA levels were analyzed as previously described [18]. Pairs with both reads fully aligned against the reference genome were filtered to keep only those mapping the plastid genome. Positions of each read were rounded down to the nearest kb and all reads mapping to the plastid large inverted repeats (IRs) were only assigned to the first IR. Two sequences of 400 bp centered on the joining of the second IR to the long and short single-copy regions were added as additional chromosomes to the reference genome to remove false positives. The Galaxy workflow "Rearrangement Junction Detection Arabidopsis Plastid" is available under Shared Data on the Galaxy server (usegalaxy.org), SCARR and other custom Python scripts used are available on GitHub (https://github.com/SamTremblay/SCARR).

### Run-on transcription assay

Chloroplasts were isolated from 2 g of leaves of 10-d-old plants grown on soil as described previously [41] with some modifications. Briefly, leaves were ground in 30 ml of cold homogenization medium (0.33 M sorbitol, 50 mM HEPES-KOH, pH 7.3, 2 mM EDTA, 0.1% BSA) using a Polytron homogenizer set on 4.5 for 5 pulses of 7 sec each. The homogenate was poured through 4 layers of Miracloth (Calbiochem), centrifuged at 4,000 g for 5 min at 4°C, and resuspended in 2 ml of cold resuspension buffer (0.33 M sorbitol, 50 mM HEPES-KOH, pH 7.5). The chloroplast suspension was loaded onto Percoll step gradients (20/70%) and centrifuged in a swing-out rotor at 7,000 g for 1h at 4°C. Intact chloroplasts were recovered at the interface of the 20% and 70% Percoll layers, washed twice with 10 volumes of cold resuspension buffer and recovered as a pellet after centrifugation at 7,000 g for 1 min. Quantification was performed using a hemocytometer and aliquots containing 10^7^ chloroplasts were used for each line. Run-on transcription assays and hybridization to dot-blot membranes were carried out as described previously [42]. As a negative control for WT chloroplasts, 1 μL of RNAse A (Fermentas) was added at the beginning of the *in vitro* transcription assay to verify that the detected signal belonged to the radioactive nucleotides incorporated in the transcripts. DNA probes were amplified by PCR using the primer pairs listed in S1 Table and 150 ng of each was fixed onto a Hybond-N+ membrane (Amersham) for hybridization.

### Quantitative PCR analysis of PSBA levels at the CREII cleavage site in 347 transgenic lines

Total DNA was isolated from 14-d-old Arabidopsis wild type (WT), 347, 347*why1why3*, and 347*sig6-1* plants grown on MS plates alone or supplemented with 10 μM estradiol, or 10 μM estradiol + 100 mg/L rifampicin, using the CTAB DNA extraction protocol [40]. Two different sets of primers harboring the CREII cleavage site were used to quantify the levels of error-free ptDNA at the psbA locus. Every reaction was carried out on biological and technical triplicates relative to the amplification of the LSC middle region (positions 45345 to 45525) in the plastid genome. Primers used for qPCR were calibrated to ensure the amplification of a unique PCR product and efficiency between 1.90 and 2.05. Primers sequences are listed in S1 Table. The Power SYBR Green PCR Master Mix (Applied Biosystems) was used according to the manufacturer’s instructions. qPCR experiments and analysis were carried out using a LightCycler 480 (Roche) and the LightCycler 480 software version 1.5, respectively.

### Quantitative RT-PCR analysis of *RNAse H1* expression in transgenic lines

Total RNA isolation and quantitative RT-PCR analysis of 14-d-old Arabidopsis plants transformed with either the RNAse H1 inducible expression vector or the empty vector (control) were performed as described previously [43]. Every reaction was carried out on biological and technical triplicates relative to the amplification of TUB5 and UBQ5. Primers used for qRT-PCR were calibrated to ensure the amplification of a unique PCR product and efficiency between 1.90 and 2.05. Primers sequences are listed in S1 Table. The Power SYBR Green PCR Master Mix (Applied Biosystems) was used according to the manufacturer’s instructions. qPCR experiments and analysis were carried out using a LightCycler 480 (Roche) and the LightCycler 480 software version 1.5, respectively.

### Western blot analysis

Chloroplasts were isolated from 14-d-old Arabidopsis plants transformed with either the RNAse H1 inducible expression vector or the empty vector (control). Chloroplast proteins were obtained as described in [44]. Equal amounts of proteins were subjected to SDS-PAGE 15% (w/v) polyacrylamide gel and blotted on a PVDF membrane (Amersham) with semi-dry transfer for 1h. The membrane was blocked with TBS-T (20 mM Tris-HCl, pH 7.6, 150 mM NaCl and 0.1% Tween 20) containing 5% nonfat milk for 1 h at room temperature and then incubated with primary antibody anti-StrepII-tag (1:1000 dilution—ProSci # 4335) overnight at 4°C. The membrane was washed with TBS-T (20 min, 3 times, at room temperature), stained with HRP conjugated anti-rabbit secondary antibody (1:10,000) for 1 h at RT, washed with TBS-T (20 min, 3 times, at room temperature), and analyzed by enhanced chemiluminescence (ECL; Amersham).

### Dot-blot analysis

For detection of DNA:RNA hybrids, chloroplast DNA was isolated from 14-d-old Arabidopsis plants (as described for run-on assays). Plastid DNA levels were equilibrated by semi-quantitative PCR using atpB as a reference gene. DNA dilutions were fixed onto a Hybond-N+ membrane (Amersham) using an ultraviolet crosslinker. Additionally, a negative control (RT reaction performed using RNA from WT plants and oligo(dT) primers treated with RNAse H) and a positive control (RT reaction not treated) were included. The membrane was blocked with TBS-T (20 mM Tris-HCl, pH 7.6, 136 mM NaCl and 0.05% Tween 20) containing 1% nonfat milk for 1 h at 4°C and incubated with S9.6 antibody (1:2500 dilution–Kerafast # ENH001) overnight at 4°C. The membrane was washed with TBS-T (20 min, 3 times, at room temperature), stained with HRP conjugated anti-mouse secondary antibody (1:10,000), washed with TBS-T (20 min, 3 times, at room temperature), and detected by ECL (Amersham).

### Chlorophyll extraction and quantification

For each line analyzed, 10 mg of plants grown on soil under normal light conditions were ground in 400 mL dimethylformamide and centrifuged for one minute at 13,000 rpm. The supernatant was recovered, and absorbance was measured at 645 nm and 663 nm. Chlorophyll content was then calculated as described previously [45].

## Results

### *sig6* mutants have reduced PEP-dependent plastid transcription efficiency and accumulate fewer ptDNA rearrangements

**Fig 1:**
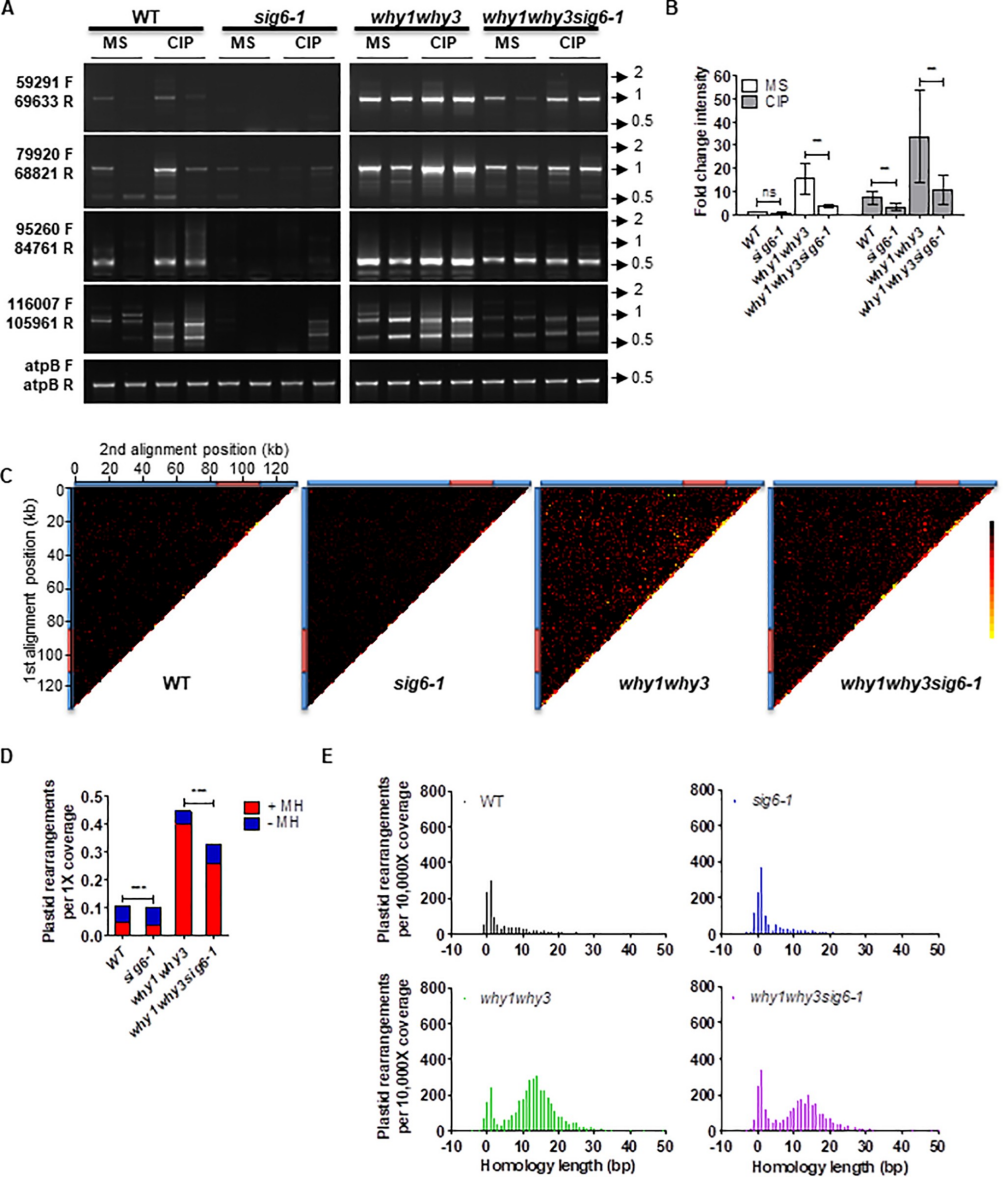
SIG6-dependent transcription induces plastid genome instability. **(A)** Representative PCR reactions from a total of 8 to evaluate the abundance of ptDNA rearrangements (number and intensity of bands represent rearranged DNA), carried out on total leaf DNA of wild-type (WT), *sig6-1*, *why1why3*, and *why1why3sig6-1* plants grown 14 days after germination (DAG) on solid MS alone or with 0.5 μM CIP. PCR experiments were performed at least three times with duplicate samples. Low cycle amplification of the atpB plastid gene was used as a loading control. Primers pairs used for PCR reactions are indicated on the left side of the gels. Arrows and numbers on the right side of the gels represent the position and size of the DNA ladder bands in kb. **(B)** The bar graph represents the fold change intensity (mean ± standard error) of the PCR bands shown in **A** for each line compared to the WT grown on MS for each primer pair (n = 8). *P*-values are calculated using a two-tailed paired t-test (ns: not-significant, **: p<0.01). **(C-F)** DNA-seq analyses of WT and mutant plants grown for 14 DAG. **(C)** Plastid rearrangement breakpoint positions of the indicated Arabidopsis mutant lines. Heat maps depict each rearrangement as the intersection of the two genomic positions corresponding to the nucleotide on each side of the junction. Each tile represents a region spanning 1 kb along each axis. Tile intensity represents the number of rearrangements normalized to the coverage per 10,000 plastid genomes (scale: 0 = black, 7.5 = red and 15 = yellow). All rearrangements mapping to the plastid large inverted repeats (IRs) were only assigned to the first IR. The plastid large-single copy region (LSC), the first IR, and the small- single copy region (SSC) are depicted as a long blue bar, a red bar and a short blue bar, respectively. **(D)** Histograms represent the total number of plastid rearrangements displaying a microhomology ≥5 bp (+MH, red) or <5 bp (-MH, blue) at their junction. All values were normalized to 1X genome coverage. P-values were generated by a χ2 test relative to the distribution of rearrangements for WT or *why1why3* plants (***: p<0.0001). **(E)** Homology usage for deletions, duplications, and inversions. Histograms represent the total number of unique events for which a homology of given length is found at the breakpoint junction. Negative homology lengths represent base insertions at the breakpoint junction. All values are normalized to the coverage per 10,000 genomes.

To evaluate the role of transcription in Arabidopsis plastid genome instability, we first analyzed the accumulation of ptDNA rearrangements in 14-d-old mutant plants for *SIG6* (line *sig6-1*). We performed a well-established semi-quantitative PCR approach using eight outward- and inward-facing primer pairs distributed throughout the plastid genome (S1 Fig) to monitor duplication/circularization and deletion events, respectively [16]. Using this approach, PCR amplification only occurs if a ptDNA rearrangement brings together the annealing sites of the primers. Plants were grown with or without ciprofloxacin (CIP), a specific inhibitor of the DNA gyrase AtGyrA [46] that induces organelle DSBs [20]. In the absence of treatment (MS), there are few ptDNA rearrangements in either wild type (WT) or *sig6-1* plants (Fig 1A and 1B). Interestingly, CIP-induced ptDNA rearrangements in WT are largely prevented in *sig6-1* plants. We then analyzed the accumulation of ptDNA rearrangements in 14-d-old mutant plants for all six Arabidopsis sigma factors required for initiation of transcription by the PEP. Notably, only mutant lines for *SIG6* (*sig6-1* and *sig6-2*) were found to accumulate lower rearrangement levels than wild type (WT) plants throughout the plastid genome in the presence of CIP (S2 Fig). Although mutants for the five other Sigma factors sometimes showed fewer rearrangements for a subset of the plastid regions analyzed, this reduction was not observed across the entire plastid genome (S2 Fig). These results are consistent with the role of SIG6 as a major general sigma factor in plastids during early plant development [10,11], and suggest that a global reduced PEP transcription at an early stage minimizes the destabilizing effects of DSBs.

We have previously shown that the *why1why3* double mutant is hypersensitive to CIP and presents high levels of ptDNA rearrangements [16,17,20]. We therefore crossed *why1why3* with *sig6-1* plants to determine the impact of reduced PEP-dependent transcription in a rearrangement-prone background. We first studied the transcription rates in 10-d-old *sig6-1* and *why1why3sig6-1* seedlings by run-on transcription blots using probes for five plastid genes: psbA, psaA, and rbcL (class I), rrn16S (class II) and accD (class III) [5]. Interestingly, we found that all the studied genes presented lower transcription rates in the absence of SIG6 at 10 days (S3 Fig). Steady-state transcript levels for psaA, psaA, rbcL and rrn16S were previously found to be lower in *sig6* seedlings at 4 days but restored to WT levels at 8 days. In contrast, accD transcript levels were shown to be higher in *sig6* at 5 days but restored to WT levels at 9 days [10]. Thus, our findings suggest that after 8–10 days, when the pale-green phenotype reverts, PEP transcription rates are still lower in *sig6-1*, even though the accumulation of transcripts is restored, and NEP activity decreases. This could be explained by increased (though less efficient) PEP transcription dependent on other sigma factor/s and a parallel decrease in NEP transcription, and/or by differential post-transcriptional regulation (e.g. RNA stability) of PEP and NEP-derived transcripts. Then, we assayed the formation of ptDNA rearrangements by the previously described PCR approach. Interestingly, combining the *sig6-1* and *why1why3* mutations led to a reduction in the levels of ptDNA rearrangements both in the absence and presence of CIP (Fig 1A and 1B). These results suggest that a general inhibition of SIG6-PEP transcription limits plastid genome instability in the absence of proteins that promote genome stability.

### Transcription-associated rearrangements occur through error-prone DNA repair

In order to analyze the effect of *SIG6* mutation on the patterns of genomic instability, we used a next-generation sequencing (NGS) approach to precisely map, characterize and quantify organelle DNA rearrangements (including duplications, deletions and inversions) in a single genome-wide experiment. In agreement with our previous results [18], while WT plants presented relatively low levels of overall ptDNA rearrangements, *why1why3* plants showed 4.2 times higher levels (Fig 1C and 1D). In accordance with the semi-quantitative PCR results, only slight differences were observed between WT and *sig6-1*. However, *why1why3sig6-1* showed a 1.4-fold decrease in rearrangements compared to *why1why3* (Fig 1C and 1D), suggesting that transcription affects genome stability in the absence of WHY1/3. Since transcription-associated DSBs were inferred to be replication dependent [21], the decreased appearance of ptDNA rearrangements could also be linked with decreased replication in the absence of SIG6. Thus, we investigated the possible impact of replication stress, defined by the slowing or stalling of replication fork progression resulting in DNA synthesis inhibition [22]. In NGS data, replication stress is associated with a progressive, directional decrease in DNA copy number along the genome [47]. We therefore compared ptDNA coverage curves for each mutant line to the WT. Results indicate that the coverage patterns of all mutant plants are similar to that of the WT plants (S4 Fig) and to those previously reported in the literature for plastid genomes [35,48,49]. These results were also confirmed using quantitative PCR measurements of ptDNA levels at three sites of the plastid large-single copy region (LSC) (S4 Fig). This suggests that replication is not affected in *SIG6* mutants, and further supports that reduced transcription in *why1why3sig6-1* limits genome instability.

While most PEP-dependent class I genes have been shown to be down-regulated in *sig6*, several NEP dependent genes presented higher expression levels [10]. To explore a possible link between transcription levels and rearrangements patterns we analyzed the number of breakpoint junctions along the plastid genome normalized to the coverage using a 100 nt window (S5 Fig). *sig6-1* accumulated somewhat similar levels of rearrangements throughout the entire plastid genome compared to WT. In turn, *why1why3sig6-1* accumulated generally fewer rearrangements compared to *why1why3*, even at rpoB/C genes, which are thought to be exclusively transcribed by the NEP and which were shown to have increased expression levels in *sig6-1*. Thus, it was not possible to distinguish precise regions of genome stability and instability associated to differential PEP and NEP transcription. Nevertheless, we did observed particular peaks of rearrangements in WT ptDNA, which significantly decreased in *sig6-1*. For example, within the rrn operon, we observed two peaks of rearrangements at trnI-intron (oriA) and trnA-intron in WT but not in *sig6-1*, as well as general decreased levels of rearrangements throughout the rrn operon in *sig6-1*. This could be explained by decreased transcription of rRNA genes in *sig6-1* [10] (S3 Fig), since it has been widely shown that highly transcribed rRNA and tRNA genes are hotspots for conflicts between replication and transcription, challenging genome integrity [50,51]. Here, we also observed peaks of rearrangements at the intergenic spacers between trnL-trnF, psbE-petL, and trnS-trnG in WT ptDNA and decreased levels in *sig6-1*. It is thought that intergenic spacers, which can contain repetitive and palindromic DNA sequences, act as replication fork barriers preventing frequent head-on collisions by slowing down or stalling the progression of replication in the direction opposite to RNAP transcription. However, if not properly regulated, they could lead to fork collapse and a consequent increase in DSBs [52,53]. Indeed, intergenic spacers at tRNA and rRNA genes, non-coding regions next to tRNAs, and some introns, were prone to accumulate higher levels of rearrangements, especially in the absence of WHY1/3 proteins, suggesting that these regions are fragile sites and that WHY1/3 protect them against illegitimate recombination. Interestingly, the absence of SIG6 was not sufficient to decrease the accumulation of ptDNA rearrangements at the rrn operon in the *why1why3* background (S5 Fig).

The elimination of DSBs proceeds via error-free homologous recombination (HR) or error-prone pathways such as microhomology-mediated recombination (MHMR) and non-homologous end-joining (NHEJ) [54]. While NHEJ typically requires microhomologies of <5 bp at the joining ends and results in small insertions/deletions (indels) of 1–4 bp, microhomologies between 5–25bp suggest the triggering of MHMR [55]. Thus, to better understand how the *sig6-1* mutation reduces ptDNA instability, we divided the observed rearrangements into two classes: those that possess a microhomology of 5 bp or more (+MH) at their breakpoint and those without (-MH). While approximately 0.048 ptDNA rearrangements per genome arose from +MH in WT plants, it decreased to 0.036 in *sig6-1* and increased to 0.40 in *why1why3* and to 0.26 in *why1why3sig6-1* lines (Fig 1D). Conversely, the level of ptDNA rearrangements per genome generated by -MH increased from 0.057 in WT plants to 0.064 in *sig6-1* and 0.066 in *why1why3sig6-1* lines, and decreased to 0.048 in *why1why3* (Fig 1D), indicating that the effect of *sig6-1* is observed mostly on +MH rearrangements. We then analyzed the length of sequence homology at the breakpoint junctions of the rearrangements to gain insight about the homology requirements for their formation. For WT and *sig6-1* datasets we observed a peak at 0–2 bases of homology, but for *why1why3* and *why1why3sig6-1* we observed another peak between 5 and 21 bases of homology (Fig 1E). Interestingly, *SIG6* mutation resulted in an increase of rearrangements with 0–2 bases of homology compared to WT, and a decrease of rearrangements produced by micro-homologies of 5–21 bases compared to *why1why3*. Thus, while the absence of Whirly proteins promoted the appearance of MH-dependent rearrangements (as previously reported in [18]), the lack of SIG6 reduced the appearance of these rearrangements and in turn, slightly increased the formation of rearrangements by MH-independent pathways.

### General and locus-specific inhibition of transcription all correlate with reduced ptDNA instability

**Fig 2:**
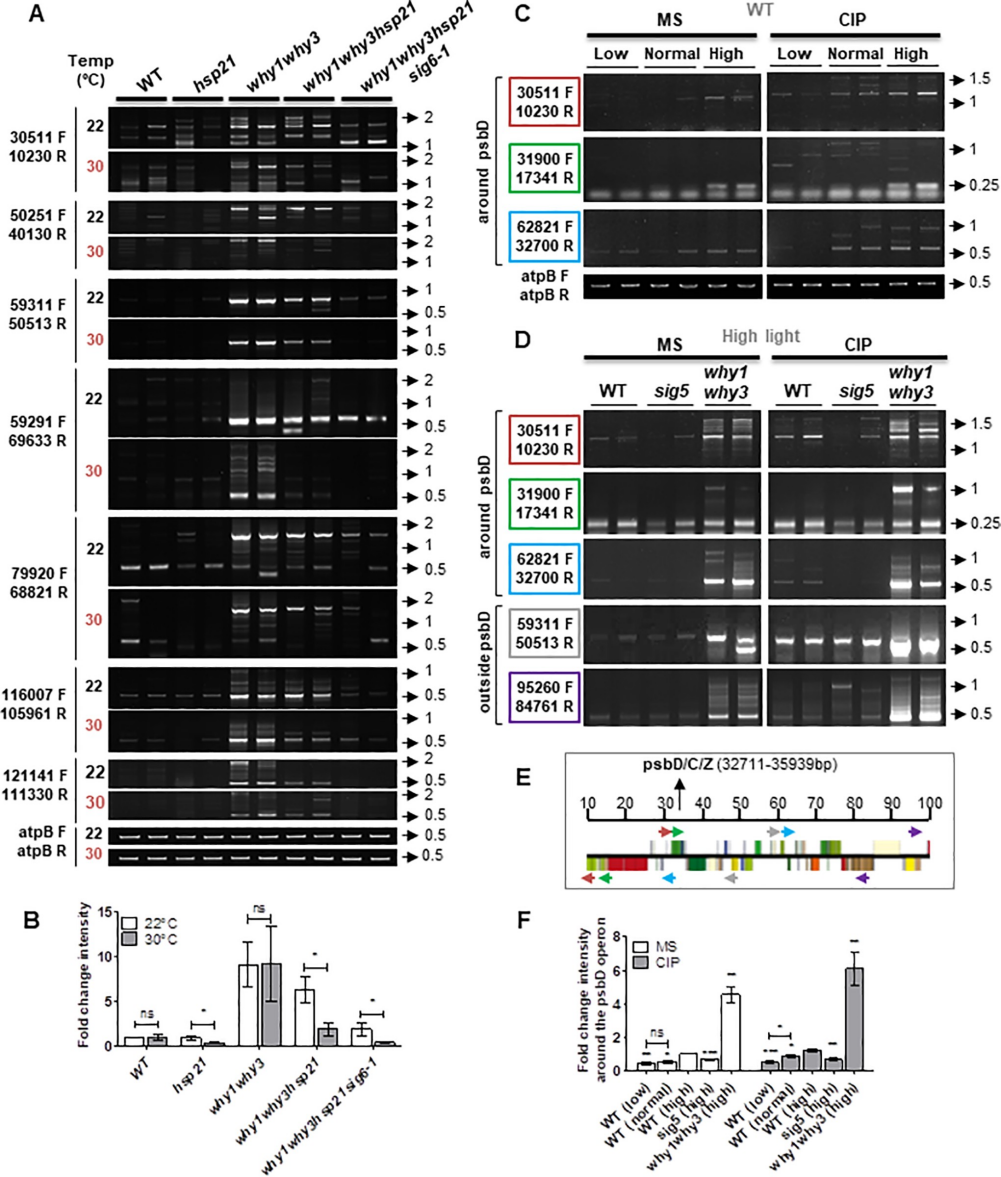
Modulation of PEP-dependent transcription affects plastid genome stability. **(A)** PCR analysis of ptDNA rearrangements in wild type (WT), *hsp21*, *why1why3*, *why1why3hsp21* and *why1why3hsp21sig6-1* plants grown in solid basal media at normal (22°C) or high temperature (30°C). **(B)** The bar graph represents the fold change intensity (mean ± standard error) of the PCR bands in **A** compared to WT for each primer pair (n = 8). **(C, D)** PCR analysis of ptDNA rearrangements in the proximity of the psbD locus in **(C)** wild-type (WT) plants grown in low, normal, or high light conditions (10, 100 or 1,000 μmol photons m–2 s–1) and **(D)** WT, *sig5* and *why1why3* plants grown in high light conditions, for 14 days on solid basal media alone (MS) or with 0.5 μM ciprofloxacin (CIP). PCR analyses outside the psbD locus were used as controls. **(E)** The image shows the plastid genetic environment of psbD operon. Colored arrows represent the primer pairs used, as indicated on the left side of the gels, following the same color code. The top line indicates the position (in kb) on the plastid genome. **(F)** The bar graph represents the fold change intensity (mean ± standard error) of the PCR bands in **C and D** compared to WT plants grown in high light conditions on MS for each primer pair (n = 3). All PCR experiments were performed at least three times with duplicate samples. Low cycle amplification of the atpB plastid gene was used as a loading control. Primers pairs used for PCR reactions are indicated on the left side of the gels. Arrows and numbers on the right side of the gels represent the position and size of the DNA ladder bands in kb. *P*-values were generated by a Two-tailed paired t-test, (ns: not-significant, *: p<0.05, **: p<0.01, ***: p<0.001).

To confirm that the effect of the *SIG6* mutation on genome instability was indeed caused by reduced transcription, and not by a secondary effect such as the lack of synthesis of a SIG6-dependent plastid-encoded protein involved in DNA metabolism, we tested the effect of other plastid transcriptional mutants on genome stability. We first used an *hsp21* mutant line that broadly represses transcription in a temperature-dependent manner. The Arabidopsis chloroplast small heat shock protein HSP21 is implicated in PEP-dependent transcription at high temperatures and its mutation leads to inhibition of transcription at 30°C, resulting in an ivory phenotype [34]. Similar to *sig6* expression patterns, *hsp21* shows decreased transcripts of PEP-dependent class I genes at 30°C, and enhanced transcripts for class III genes (including rpoA and rpoB) compared with the WT [34]. Our results indicate that *hsp21* mutant plants do indeed have reduced ptDNA rearrangements levels at 30°C (Fig 2A and 2B). Moreover, when we crossed *why1why3* plants with *hsp21* and *sig6-1* plants, we observed that triple mutant *why1why3hsp21* plastids also accumulated fewer rearrangements at 30°C compared to the *why1why3* double mutant. This effect was even more pronounced in the *why1why3hsp21sig6-1* quadruple mutant (Fig 2A and 2B), indicating that reduced transcription in *hsp21* lines under heat stress might limit ptDNA instability. Noteworthy, we did not observe an increased accumulation of ptDNA rearrangements in WT and mutant plants grown at 30°C compared to 23°C. This is not completely unexpected because on one side, heat stress induces replication-dependent DSBs, but on the other side, it can also cause inhibition of transcription, arrest or deceleration of the progression of the replication forks, and inhibition of DNA repair [56], Thus, although we cannot exclude a role of HSP21 in ptDNA replication or repair, which would impact the accumulation of rearrangements associated with transcription-replication conflicts, our results suggest that modulation of PEP-dependent transcription also impacts the appearance of ptDNA rearrangements.

To further test the specificity of transcription on the observed ptDNA rearrangements, we analyzed the impact of the sigma factor SIG5 on the accumulation of rearrangements around the psbD locus. SIG5 specifically activates the transcription of the psbD blue light-responsive promoter (BLRP) upon high light stress [57,58]. First, WT plants were grown under low, normal and high light conditions and assayed for the formation of plastid rearrangements in the proximity of the psbD locus. Semi-quantitative PCR results show a direct correlation between light intensity and rearrangement levels near the light-induced transcription locus (Fig 2C–2E and 2F). To precisely characterize these rearrangements, the major induced PCR products were excised from gels for direct sequencing. Results are summarized in Table 1, which indicates the mapped junction sequences and the presence of microhomology repeats. In the absence of SIG5, psbD*-*BLRP transcription is abolished [58]. Interestingly, we observed that in the *sig5* mutant, the appearance of rearrangements induced in WT plants grown under high light is strongly inhibited in the proximity of the *psbD* locus either in the absence or presence of CIP-induced DNA damage (Fig 2D, 2E and 2F). In contrast, rearrangement levels did not seem to be affected in other regions of the plastid genome. Taken together, our results convincingly show a locus-specific direct correlation between transcription levels and ptDNA instability.

**Table 1:** Characteristics of the amplified rearranged fragments formed around the psbD locus upon high light induction.

Primer pair	Repeat position*	Repeat annotation^	Short direct repeat sequences and junction	Repeat length (mismatches)
30511 F 10230 R	9928…9947	NCS between trnR-AGA and atpA	AGAATAATCC**GTTTCGTTTTTTATACTTTC**TCCTGAAGTA	(0)
	recombinant		AGAATAATCC**GTTTCG—TTTTATACTTTC**CTTGATAGAT	20 bp
	30860…30881	NCS between trnE-GAA and trnT-ACC	AGAGGGTTAA**GTTTCGTTTTTTTTTTACTTTC**CTTGATAGAT	(3)
31900 F 17341 R	17175 …17185	rpoC2	AATTGTAATT**AGAGTATTTTT**TTTTATTGAT	(0)
	recombinant		AATTGTAATT**AGAGTATTTTT**GCAAAGTAAT	11 bp
	31907…31917	NCS between trnT-ACC and psbD	CTAGAGAAAG**AGAGTATTTTT**GCAAAGTAAT	(0)
62821 F 32700 R	32290…32299	NCS between trnT-ACC and psbD	CCCGTCAACT**AAAAAAAGGG**TATAAAAGGA	(0)
	recombinant		CCCGTCAACT**AAAAAAAGGG**GGAACCATAAA	10 bp
	62953…62962	spacer between petA and psbJ	ACACGCGCCG**AAAAAAAGGG**GGAACCATAAA	(0)

* Indicates the positions of the short direct repeats (shown in bold) referred to the number of the nucleotides in the *A*. *thaliana* chloroplast genome sequence.

^ Indicates the annotation of the direct repeat within the coding or non-coding sequence (NCS) of the chloroplast genome.

Finally, to confirm that this effect of transcription on plastid genome stability is not confined to genes transcribed by the PEP, we also evaluated the effect of reduced NEP-dependent transcription on ptDNA stability. We used mutant plants lacking a nuclear gene that encodes the plastid RPOTp RNA polymerase (NEP) required for chloroplast biogenesis [59]. *rpotp* mutant plants grown on CIP presented fewer rearrangements than WT plants grown on the same media (S6 Fig).

All in all, these results indicate that both PEP- and NEP-dependent transcription affect plastid genome integrity.

### Reduced psbA transcription prevents rearrangements caused by DSB-induced microhomology-mediated repair

We have previously shown that high levels of DSBs induced by CIP promote the accumulation of rearrangements throughout the plastid genome, especially in plants lacking Whirly proteins, which accumulate higher levels of MHMR rearrangements than the WT controls [17]. Sequencing results presented here suggest that transcription also promotes the formation of ptDNA rearrangements through error-prone DNA repair mechanisms. Thus, we evaluated the impact of transcription of psbA, which is a highly expressed plastid gene [60], on DSB repair using the transgenic line 347 developed by Kwon et al. (2010) [36]. Importantly, the psbA gene is transcribed from a single PEP promoter; its expression increases markedly at the early stage of chloroplast development [61] and is differentially maintained in a light-dependent manner even when the total plastid transcription declines as a consequence of chloroplast maturation [62]. Upon β-estradiol induction, line 347 expresses a plastid-targeted endonuclease I-CREII, which cleaves the endogenous psbA gene at a specific site. DNA sequencing of rearrangements formed around this break site have confirmed that they were mostly generated by MHMR [36].

**Fig 3:**
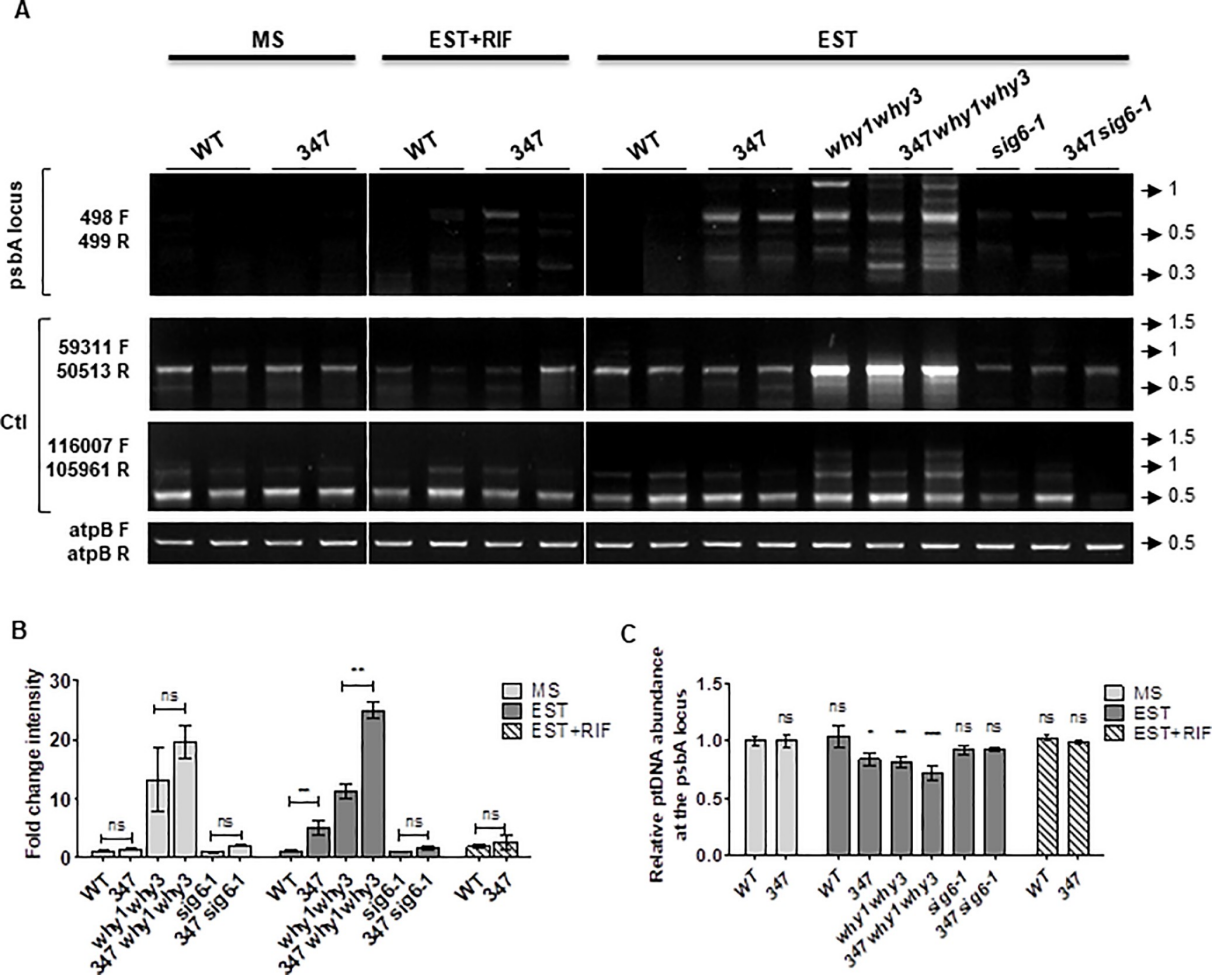
ptDNA rearrangements are induced by a single DSB in Arabidopsis transgenic line 347 and prevented by transcription inhibition. **(A)** PCR analysis at the psbA locus with primers 498 and 499 on total leaf DNA of wild type (WT), *I-CREII* expressing line 347, *why1why3*, 347*why1why3*, *sig6-1* and 347*sig6-1* plants grown for 14 days on solid basal media (MS, non-induced), with 10 μM β-estradiol (EST, induced), or with 10μM β-estradiol plus 100 mg/L rifampicin (EST+RIF, induced). All PCR experiments were performed at least three times with duplicate samples. PCR amplifications using primer pairs mapping outside the psbA locus were used as controls (Ctl). Low cycle amplification of the atpB plastid gene was used as a loading control. Primers pairs used for PCR reactions are indicated on the left side of the gels. Arrows and numbers on the right side of the gels represent the position and size of the DNA ladder bands in kb. **(B)** The bar graphs represent the fold change intensity (mean + standard error) of the PCR bands shown in **A** for each plant line grown on MS, EST or EST+RIF. Two-tailed paired t-test, (ns: not-significant, **: p<0.01). **(C)** Mean relative ptDNA levels at the I-CREII restriction site in the psbA locus measured by qRT-PCR in the plants described above grown for 14 days on MS, EST or EST+RIF, normalized to the abundance of ptDNA at the LSC region using primers 45345 F and 45525 R. The values were acquired from three independent experiments, and the abundance level of WT in MS was adjusted to 1. Error bars represent the standard error. One-way ANOVA and Tukey t-test (ns: not-significant, *: p<0.05, **: p<0.01, ***: p<0.001).

Here, to assert the effect of transcription on the accumulation of rearrangements upon induction of one single and specific DSB in the plastid genome, we performed semi-quantitative PCRs using primers located ~5 kb apart and encompassing the I-CREII cleavage site (named 498 and 499). We first validated this approach by confirming that plants of the 347 transgenic line grown on MS medium alone (without induction) did not accumulate genomic rearrangements around the break site, whereas β-estradiol (EST) induction of one DSB per plastome copy was sufficient to increase the appearance of rearrangements specifically at the psbA locus (Fig 3A and 3B). Moreover, when I-CREII was expressed in the *why1why3* background, the level of rearrangements around the psbA locus increased. This is consistent with previous reports showing that Whirly proteins prevent MHMR [17,18]. In contrast, when I-CREII was expressed in reduced psbA transcription conditions, i.e. in the *sig6-1* mutant background or in the WT background in combination with a mild RIF treatment, these genomic rearrangements became undetectable (Fig 3A and 3B). These results suggest that either transcription acts as an obstacle to conservative repair or it is required for MHMR pathways. To discern between these two possibilities, we performed qPCR assays to quantify the relative amount of error-free repaired plastid copies at the I-CREII restriction site (Fig 3C). We verified that in line 347, I-CREII induction significantly reduced the abundance of ptDNA harboring the intact restriction site, and that either in the absence of SIG6 (line 347*sig6-1*) or after treatment with RIF, the abundance levels were similar compared to WT plants. Collectively, our results support the hypothesis that reduced transcription rates facilitate the conservative repair of DSBs.

### Loss of SIG6 partially rescues the phenotypes of mutants with high ptDNA instability

**Fig 4:**
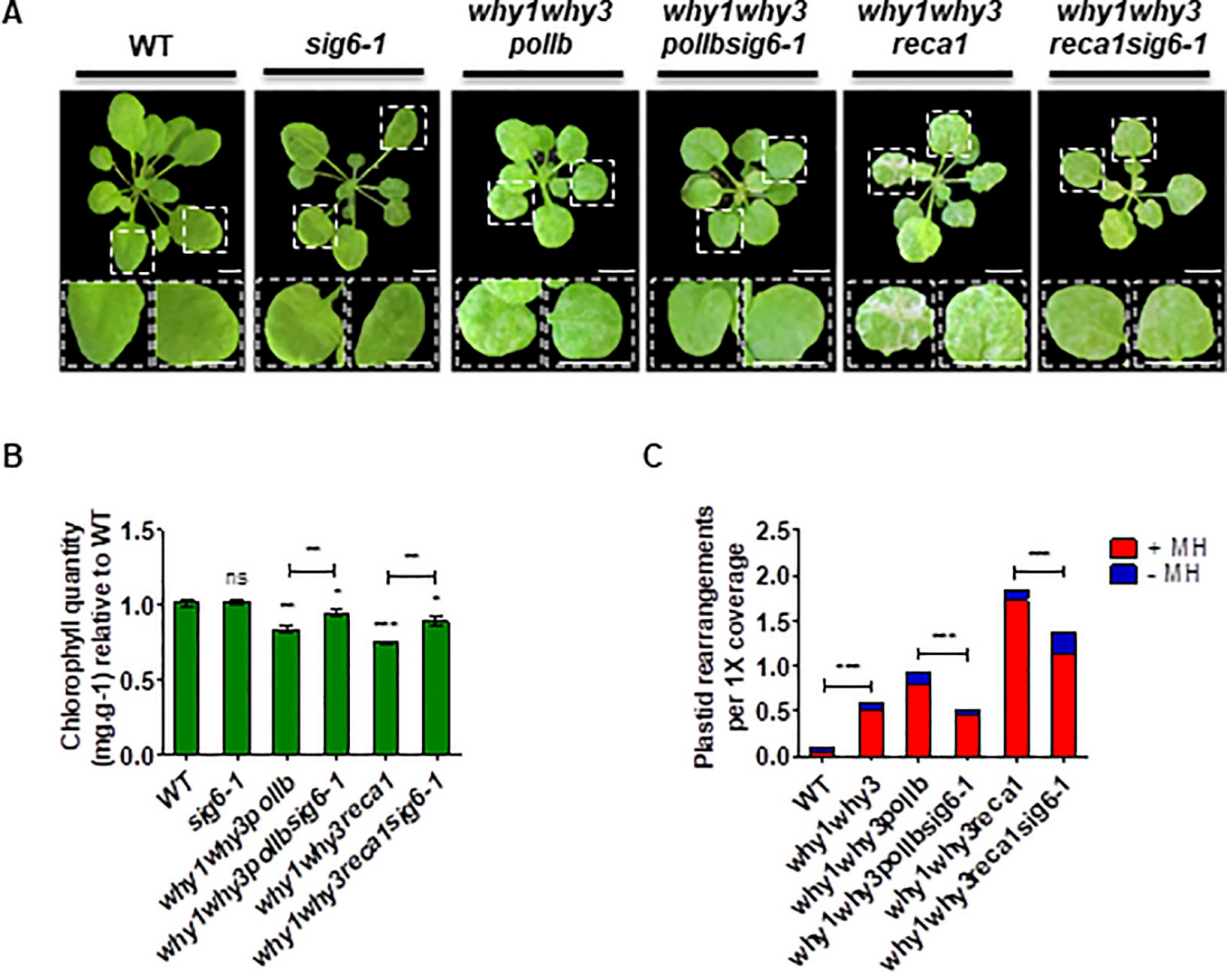
*SIG6* mutation partially complements *why1why3polIb* and *why1why3reca1* phenotypes. **(A)** Representative photographs of Arabidopsis wild type (WT), *why1why3polIb*, *why1why3polIbsig6-1*, *why1why3reca1*, *why1why3reca1sig6-1*, and *sig6-1* mutant plants grown 28 days on soil. Bar = 0.5 cm. **(B)** Histogram showing the quantification of chlorophyll a and b (mean ± standard error) of plants described in **A** relative to WT (n = 6, 10, 10, 10 and 6, respectively). Mann Whitney test, (ns: not significant,*: p<0.05, **: p<0.01, ***: p<0.001). **(C)** DNA-seq analysis of WT, *why1why3*, *why1why3polIb*, *why1why3polIbsig6-1*, *why1why3reca1*, *why1why3reca1sig6-1* grown on soil for 14 days after germination. Plastid rearrangements displaying a microhomology ≥5 bp (+MH, red) or <5 bp (-MH, blue) at their junction. All values were normalized to 1X genome coverage. χ2 test (***: p<0.0001).

To further analyze the correlation between decreased transcription-associated damage and decreased ptDNA rearrangements, we investigated the possibility that the *sig6-1* mutation could also rescue plant growth phenotypes caused by plastid genome instability. To this end, we crossed *sig6-1* plants with triple mutant *why1why3polIb-1* and *why1why3reca-1* plants, which both display white/yellow variegated leaves as a result of poorly functional chloroplasts caused by high levels of ptDNA non-homologous recombination [18] (Fig 4A). Strikingly, DNA-seq analysis of 14 day-old plants showed that the obtained quadruple mutants present much lower levels of ptDNA rearrangements as well as increased levels of chlorophyll a and b, as compared to the triple mutants (Fig 4B and 4C). This partial rescue of variegation phenotypes further supports the hypothesis that transcription-related rearrangements pose an important threat to genome stability, particularly in the absence of adequate DNA repair and maintenance machinery.

### Transcription-associated R-loops lead to genomic instability

**Fig 5:**
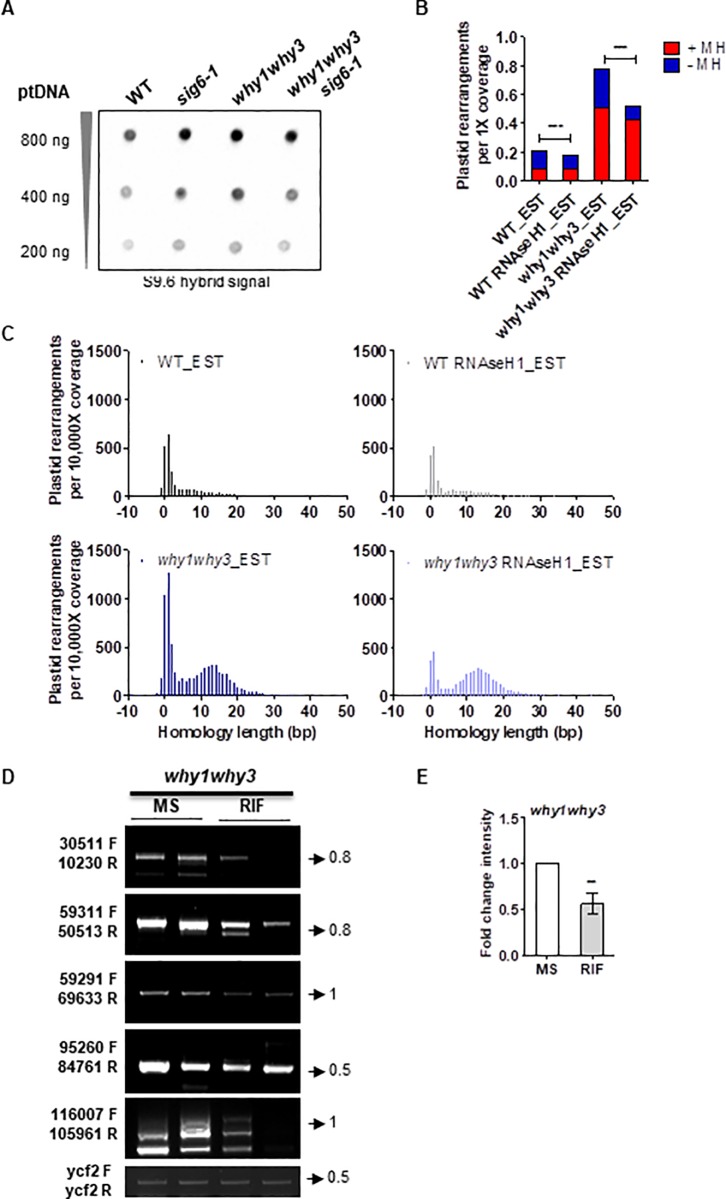
R-loops constitute a major threat to plastid genome stability. **(A)** Representative dot-blot showing the effect of *SIG6* mutation on the accumulation of plastid R-loops. Plastid DNA was extracted from chloroplast of wild type (WT) and mutant plants grown for 14 days after germination (DAG) on soil, and serial dilutions were spotted and probed with the S9.6 antibody. Experiments were carried out on biological triplicates. **(B-C)** DNA-seq analysis of organelle rearrangements in wild type (WT), *why1why3*, and transgenic plants expressing RNAse H1_A in the WT and *why1why3* backgrounds grown 14 DAG on soil and induced with 100 μM β-estradiol. **(B)** The histogram represents the total number of plastid rearrangements displaying a microhomology ≥5 bp (+MH, red) or <5 bp (-MH, blue) at their junction. Data were normalized to 1X genome coverage. χ2 test (***: p<0.0001). **(C)** Homology usage for deletions, duplications, and inversions. Histograms represent the total number of unique events for which a homology of given length is found at the breakpoint junction. Negative homology lengths represent base insertions at the breakpoint junction. All values are normalized to the coverage per 10,000 genomes. **(D)** Representative PCR reactions from a total of 8 to evaluate the abundance of ptDNA rearrangements in *why1why3* plants grown 14 days on solid media basal (MS) or containing 100 mg/L rifampicin (RIF). **(E)** The bar graph represents the fold change intensity (mean ± standard error) of the PCR bands shown in **D** for the plants treated with RIF compared to MS, for each primer pair (n = 8). Two-tailed paired t-test, (ns: not-significant, **: p<0.01). All PCR experiments were performed at least three times with duplicate samples. Low cycle amplification of the ycf3 plastid gene was used as a loading control. Primers pairs used for PCR reactions are indicated on the left side of the gels. Arrows and numbers on the right side of the gels represent the position and size of the DNA ladder bands in kb.

Since R-loops are an important link between transcription and genome instability, it is possible that in a DNA damage prone background, reduced transcription leads to fewer R-loops, and hence fewer genomic rearrangements. To test this hypothesis, we first detected the abundance of RNA:DNA hybrids in ptDNA by dot-blot analysis using the monoclonal antibody S9.6 [63] (Fig 5A). *why1why3* plastids accumulated more RNA:DNA hybrids compared to WT, which could suggest that Whirly proteins prevent the formation of R-loops in the plastid genome. Furthermore, *why1why3sig6-1* accumulated fewer RNA:DNA hybrids compared to *why1why3*, supporting the hypothesis that reduced transcription results in fewer R-loops in the absence of plastid Whirly proteins. However, Fig 5A also indicates that *sig6-1* alone accumulated more RNA:DNA hybrids compared to WT.

The RNase H family of enzymes, which degrades the RNA moiety of an RNA:DNA hybrid, plays a crucial role in resolving R-loops in eukaryotes and prokaryotes. Recently, Yang et al., (2017) found that RNH1C is responsible for plastid R-loop homeostasis and genome integrity in *A*. *thaliana* [27]. Thus, we further studied the impact of R-loops on plastid genome instability using *A*. *thaliana* transgenic lines expressing estradiol-inducible plastid-targeted *E*. *coli* RNAse H1.Three independent transgenic lines (RNAse H1_A, B and C) were selected by qRT-PCR and Western blot, and RNAse H1 activity was essayed by dot-blot detection of R-loops, taking advantage of the anti-DNA:RNA hybrid S9.6 antibody (S7 Fig). Interestingly, in *why1why3* plants, expression of exogenous RNAse H1 was able to reduce the levels of ptDNA rearrangements, either in the presence or absence of CIP, as well as the white variegation phenotype observed in seedlings after CIP treatment (S7 and S8 Figs).

Few studies have investigated how R-loop-induced damage is repaired and if defects in repair contribute to the genome instability [64]. Thus, we performed DNA-seq analyses of EST-treated 14-d-old WT and *why1why3* seedlings expressing RNAse H1 (or an empty vector) to assess whether specific pathways are involved in the repair of R-loop induced damage by analyzing the distribution of homology lengths at the junctions of rearrangements. We found that *why1why3* plastids accumulated 5.5 times more +MH rearrangements in respect to WT plastids (as previously observed in Fig 1D), that RNAse H1 expressing lines presented a global reduced accumulation of rearrangements throughout the plastid genome (S5 Fig), and that their reduced instability was mainly explained by a 1.3 and 2.7-fold reduction of -MH rearrangements compared with their respective WT and *why1why3* backgrounds (Fig 5B). This shift was also confirmed by a more detailed analysis of the lengths of the homologies leading to rearrangements. Indeed, we observed that the expression of RNAse H1 was able to significantly reduce the peak for rearrangements at 0–2 bases of homology both in the presence and absence of Whirlies, but did not affect the generation of rearrangements by microhomologies between 5 to 21 bases (Fig 5C). These results suggest that RNAse H1 prevents R-loop mediated rearrangements originated by NHEJ and that Whirly proteins mainly limit the formation of rearrangements by MHMR.

Further support for the role of transcription on ptDNA damage was obtained by measuring rearrangement levels in *why1why3* plants treated with rifampicin (RIF). By preventing transcription elongation when the transcript becomes 2 to 3 nt in length, RIF is also expected to reduce the formation of R-loops, and thus of ptDNA rearrangements. Interestingly, *why1why3* plants show fewer rearrangements when treated with RIF (Fig 5D and 5E), further suggesting that in the absence of Whirly proteins, transcriptional R-loops can result in loss of plastid genome integrity.

## Discussion

**Fig 6:**
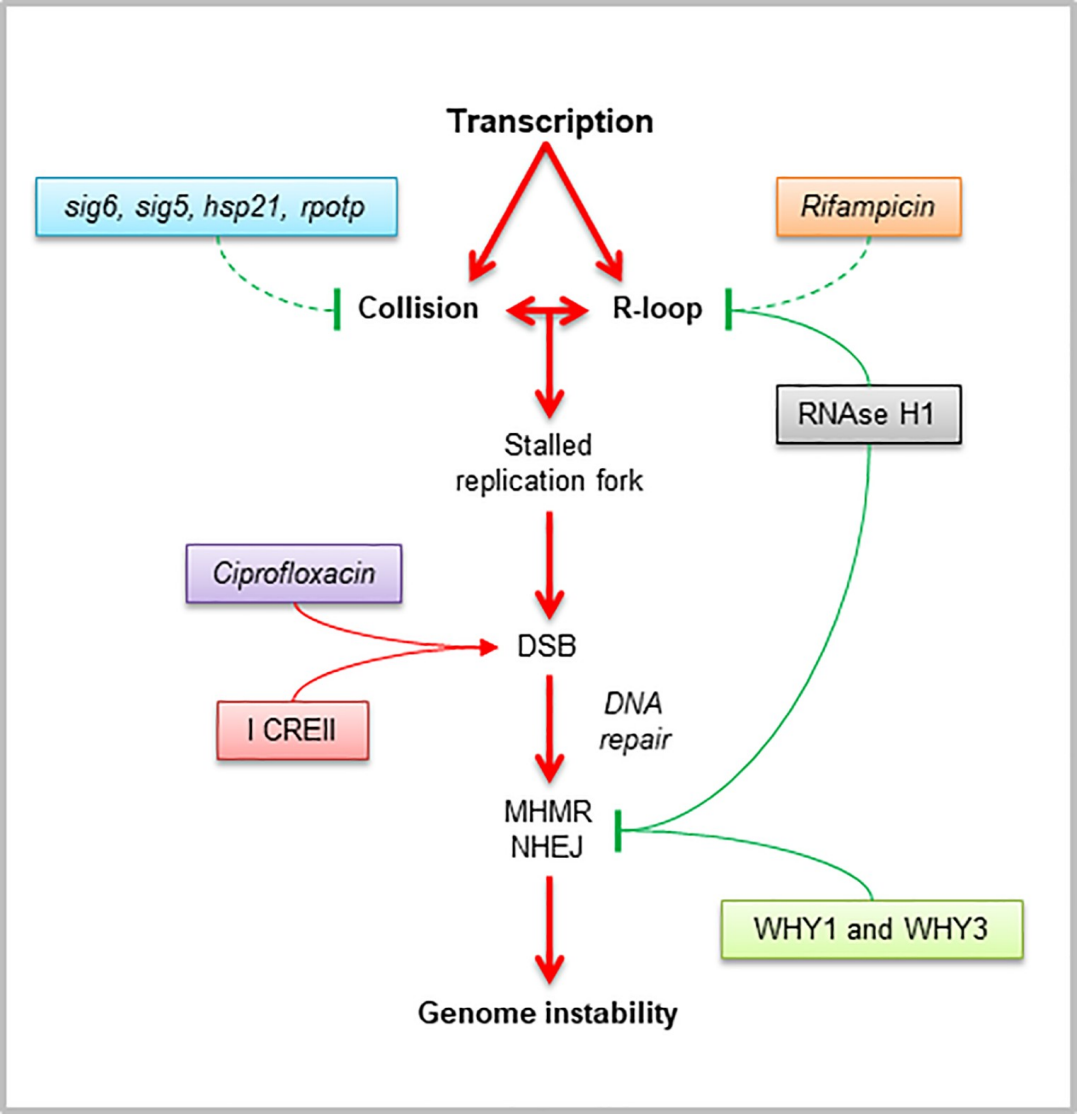
Proposed model for transcription-associated genome instability in plastids. Transcription-driven genome instability could be explained by a possible stalling of the replicating DNA polymerase when colliding with a blocked RNA polymerase or a stable DNA:RNA hybrid or by a major susceptibility of the displaced ssDNA in R-loops to DNA damage. Then, a collapsed replication fork or a DNA lesion could subsequently generate a double-stranded break (DSB), which in the presence of Whirly proteins (WHY1 and WHY3) could be repaired by error-free mechanisms before replication restart. Conversely, in the absence of Whirly proteins or in the presence of high DSB levels (induced throughout the entire plastid genome by ciprofloxacin treatment or at a specific locus by expression of the exogenous endonuclease I-CREII), a DSB could be repaired mainly by micro-homology-mediated repair (MHMR) and secondly by non-homologous end-joining (NHEJ), leading to genomic rearrangements. Plastid genome instability can be ameliorated by transcription impairment, possibly by reducing collisions between the replication and transcription machineries (*sig6*, *sig5*, *hsp21*, and *rpotp* mutants), as well as reducing the formation of R-loops (*why1why3sig6* mutants, treatment with rifampicin, expressing an exogenous plastid-targeted RNAse H1). Full lines represent pathways corroborated in this study and dashed lines show possible pathways according to the literature. Red arrows represent pathways leading to genome instability, whereas blunt end green lines show pathways promoting stability.

We have previously shown that replication stress can induce rearrangements in the plastid genome [18], leading to a severe photosynthetic electron transport chain (PET) imbalance and elevated ROS production, triggering plastid-to-nucleus retrograde signaling pathways to reprogram the nuclear transcriptome [43]. Here, we show that transcription is also a natural source of rearrangements and instability in the plastid genome, especially after induced DNA damage (Fig 6).

### Decreased plastid transcription prevents the accumulation of MHMR rearrangements

Transcription is in itself a source of DSBs. Several types of transcription stress can affect progression of the transcription machinery, and the transient strand separation during transcription renders the non-transcribed strand particularly vulnerable. In addition, transcription can impede replication progression by collisions with DNA-bound RNA polymerases (either transcribing or arrested) and with R-loops [65]. Our observations in this study show that decreased PEP or NEP activity parallels decreased formation of rearrangements in the plastid genome, possibly explained by decreased transcription-replication conflicts. Supporting this hypothesis, it was recently shown in *E*. *coli* that a large number of upregulated transcription factors promote DNA damage and mutations by replication stalling [66]. Interestingly, the authors observed that mutational ablation of the DNA-binding domains of three studied transcription factors abolished the induction of SOS DNA-damage and fork reversal foci. Since reversed forks block resumption of replication and lead to fork breakage [67], the authors proposed a model in which DNA-bound transcription factors create replication roadblocks causing fork stalling and reversal leading to DNA instability. In *E*. *coli*, it was also demonstrated that R-loops are associated with backtracked (arrested) RNA polymerases and the generation of DSBs by co-directional collision with the replisome [68]. Likewise, we can propose that in plastids, competing PEP and NEP polymerases might lead to backtracked elongation complexes promoting collisions and R-loops, and replication-dependent genome instability. This implies that maintaining a hybrid transcription system enables a rapid and coordinated chloroplast-nucleus regulation of transcription, at the expense of potential transcription-replication conflicts.

In microorganisms and mammalian cells, transcription represents a threat to genome integrity and increased transcription rates correlate with increased transcription-associated mutagenesis and recombination (reviewed in [69,70]). Here, we showed that the global impairment of either PEP or NEP general plastid transcription (i.e. *sig6* and *rpotp* mutants) resulted in reduced levels of plastid rearrangements after genotoxic treatment with CIP (Fig 1A and 1B and S6 Fig). In addition, using a next-generation sequencing approach, we corroborated that the loss of SIG6 was able to reduce the accumulation of ptDNA rearrangements generated by microhomologies of 5–21 bases in the absence of plastid surveillance factors WHY1 and WHY3 (Fig 1). This reduced accumulation of MHMR rearrangements in *why1why3sig6-1* might be explained by general decreased transcription rates (S3 Fig) and R-loops (Fig 5A).

Like their bacterial counterparts, plant sigma factors are thought to direct the PEP polymerase complex to its cognate promoters and ensure faithful transcription initiation [71,72]. Interestingly, only mutants of sigma factor SIG6, which has a primary role in transcription of most PEP-dependent genes during early development, showed reduced levels of rearrangements throughout the plastome after CIP treatment (Fig 1A and 1B and S2 Fig). In contrast, for the other sigma mutants (*sig1-sig5*), only some of the studied regions of the plastome presented fewer rearrangements at 14 days (S2 Fig). This could be explained by the fact that, although most of the plastid sigma factors showed functional overlap on many promoters, each appears to have an important and specific function for a set of promoters and for a specific metabolic pathway [7]. The mutation of these sigma factors would therefore mostly affect specific loci in the genome, as observed in the *sig5* mutant for the psbD locus upon light stress (Fig 2C–2F). Additionally, temperature modulation of transcription in *hsp21* mutants either in the presence or absence of Whirly proteins also suggests that a general decrease of PEP-dependent transcription might be responsible for lower levels of ptDNA rearrangements (Fig 2A and 2B). While HSP21 has been shown to be preferentially required for transcription by PEP rather than mRNA processing and translation [73], we cannot exclude the possibility that it might be involved in other functions (e.g., DNA replication, inheritance, repair), which would affect the generation of rearrangements.

### Plastid transcription correlates with the appearance of R-loops and genomic instability

Under conditions of replication stress, collisions of the replication machinery with the transcription apparatus may lead to the stabilization of R-loops, thereby inducing the formation of DSBs and chromosomal translocations [25]. Moreover, it was recently shown in yeast that persistent R-loops interfere with HR-mediated DSB repair, and proposed that RNAse H would remove RNA:DNA hybrids both before and after R-loops induce DSBs [64,74].

In our study, the total number of rearrangements per genome was similar in *sig6-1* and WT plastids. However, *sig6-1* accumulated comparatively less rearrangements generated by +MHs and more rearrangements generated by -MHs. This shift might be explained by a global reduction of PEP transcription dependent on SIG6 (S3 Fig), at the expense of a concomitant increase of spurious or abortive PEP transcription (dependent on other sigma factors) as well as an increase of NEP transcription from upstream SOS promoters to compensate for the loss of SIG6 [75], which results in the reversion of the pale-green phenotype at 10 days. Thus, the global decrease of PEP-dependent transcription for class I genes (e.g. rbcL, psbA, psbB, psbC, psbD, psbH, psbN and psbT) and class II rRNA genes (rrn16, rrn23, rrn5 and rrn4.5) [10] might potentially prevent DSBs caused by collisions with the NEP and replication machinery, reducing the accumulation of MHMR rearrangements in *sig6-1*. In turn, increased spurious transcripts, which are not translated, may explain the observed increased accumulation of R-loops and NHEJ dependent rearrangements (Figs 1 and 5A). Noteworthy, increased RNA-DNA hybrids in *sig6-1* may also be explained either by increased NEP-dependent transcription for some class II genes (e.g. clpP, rps15, ndhB, ycf1) and class III genes (e.g. rpoB, rpoC1, rpoC2) [10], by differential stability or processing of transcripts initiated from SOS NEP promoters, by decreased translation as a result of reduced rRNA transcription [10], as well as by the role of R-loops in the regulation of gene expression [23] and DNA replication initiation [76]. Additionally, the S9.6 antibody was shown to exhibit highly variable binding affinities towards different R-loop sequences [77], and also to detect RNA-RNA hybrids in fission yeast [78], which may impact the accurate quantification of R-loops. Thus, further studies are needed to produce a comprehensive view of the effect of *SIG6* mutation with regards to the formation of R-loops. Interestingly, the loss of SIG6 in the absence of Whirly proteins was shown to decrease the levels of plastid R-loops and rearrangements (Figs 1 and 5A), possibly as a consequence of general decreased transcription rates in *why1why3sig6-1* (S3 Fig). This would also imply that PEP-dependent spurious transcription may be impaired in the absence of both SIG6 and plastid Whirly proteins, and thus, it will be interesting to study whether a genetic interaction exists between SIG6 and WHY1/3. Likewise, in *why1why3* plastids, expression of an exogenous plastid-targeted RNAse H1 and transcription inhibition with RIF, both correlated with reduced levels of plastid rearrangements, suggesting that R-loops impair error-free DNA repair in plastids (Fig 5B–5E, S7 and S8 Figs). Our results are therefore consistent with a model in which proper replication and repair are essential for mitigating R-loop-induced genome instability [79]. This is further supported by the partial complementation of the *why1why3polIb* and *why1why3reca1* phenotypes by the *sig6-1* mutation (Fig 4). Remarkably, while *SIG6* mutation reduced the appearance of MHMR rearrangements, the expression of RNAse H1 mainly restricts the appearance of NHEJ. This implies that before the formation of RNA-DNA hybrids, DSBs would be more prone to form rearrangements through MHs, whereas after their formation, they would be preferentially repaired by non-homologous pathways (as MHs would be less available to anneal illegitimately).

### Transcription competes with ptDNA repair

Results obtained with the 347 transgenic lines expressing the plastid-target endonuclease I-CREII revealed that PEP transcription inhibition by *SIG6* mutation or treatment with RIF prevented the appearance of rearrangements caused by I-CREII cleavage at the psbA locus (Fig 3A and 3B). Considering that most of these rearrangements are generated by MHMR mechanisms [36], one possible hypothesis is that transcription may be necessary for MHMR pathways. Different transcription-coupled DNA repair (TCR) pathways have been described to maintain genome integrity, but to date, there is no direct evidence linking transcription to microhomology-dependent repair [80]. Nevertheless, emerging evidence indicates that an RNA-templated transcription-associated recombination mechanism is important to protect coding regions from DNA damage-induced genomic instability [81–83]. Recently, genetic studies in yeast have shown that RNA transcripts cooperate with RAD52 to coordinate homology-directed DNA recombination and repair in the absence of a DNA donor, demonstrating a direct role for transcription in RNA−DNA repair [84]. Alternatively, transcription could interfere with conservative DNA repair, especially under conditions of replication stress. Indeed, DNA breaks stimulate DNA–RNA hybrid formation and must be subsequently removed to allow DSB processing and repair [85]. In line with this hypothesis, we observed that both the line 347 in the *sig6-1* background and the line 347 treated in combination with RIF, had similar error-free repaired ptDNA abundance levels at the psbA restriction site compared to WT plants (Fig 3C), suggesting that decreased global levels of PEP transcription enable conservative DNA repair.

### Transcription-replication conflicts generate ptDNA instability

In plastids, we propose a model in which R-loops act as a roadblock not only for oncoming RNA polymerases, but also for the advancing replication fork, and thus constitute a major cause of transcription-replication conflicts that can generate DSBs [21] and ultimately trigger plastid genome instability (Fig 6). Replication forks can be blocked and collapsed by R-loops if they collide with unrepaired DNA lesions in the ssDNA displaced strand, with the RNA:DNA hybrid itself, or with an RNA polymerase (RNAP) putatively trapped at the transcription site by the R-loop [86]. Prolonged stalled forks may be processed into DSBs by structure-specific endonucleases that generate DSBs and promote error-prone break-induced replication in an attempt to resume DNA synthesis. DSBs may also arise by endonucleases or passive breakage of persistent ssDNA [22]. In the presence of Whirly proteins, a DSB can be subsequently repaired through conservative repair mechanisms such as homologous recombination (HR), with RECA1 and POLIB proteins playing an important role to restart the replication fork without compromising genome integrity [18]. Conversely, in the absence of plastid Whirly proteins or in the presence of high levels of DSBs induced by CIP, DNA-repair is more likely to occur by error-prone mechanisms such as MHMR and NHEJ pathways. These observations are in line with recent studies pointing to microhomology-mediated end joining (MMEJ) as the principal mediator of DSB repair during mitochondrial DNA lesions in humans [87]. Interestingly, replication protein A (RPA) was shown to prevent MMEJ in yeast, possibly binding to the ssDNA overhangs to impede spontaneous annealing between microhomologies [88], and to function as a sensor of R-loops and a regulator of RNAse H1 in human cell lines [89]. In Arabidopsis, it was shown that WHY1/3 co-purified with RNH1C [27], and here, we showed that *why1why3* plastids accumulate more R-loops than the WT, possibly suggesting a common role for ssDNA-binding proteins in suppressing R-loops and the associated genomic instability (Fig 5A). The observed links among plastid transcription, R-loops and Whirly proteins, raise the interesting question of whether WHY1/3 is a sensor of R-loops. Alternatively, since in maize WHY1 was found to bind in vivo with a subset of plastid RNAs, promote atpF intron splicing and influence the biogenesis of the large ribosomal subunit [90], we can also propose that Whirly proteins might prevent R-loop formation by its role in RNA processing. To address this issue, further research employing high-resolution and strand-specific techniques such as DRIPc-seq to quantify and map R-loop structures plastome wide as well as rRNA biogenesis studies are needed.

Taken together, our results suggest that reducing plastid transcription may contribute to diminish the frequency of collisions between NEP and PEP transcription machineries and also with the replisome and prevent the accumulation of DNA-damaging R-loops in the absence of Whirly proteins or in the presence of genotoxic stress. In support of this idea, most mechanisms to avoid transcription-associated conflicts in bacteria involve factors that destabilize or remove RNAP from the template, allowing a clearer path for the replisome [91]. In plastids, transcription is controlled by synthesis/degradation of PEP and NEP, and also by stimulation/inhibition of their activities (reviewed in [4]). It is therefore possible that in plastids, modulation of PEP and NEP may alleviate replication-transcription conflicts or resolve them by favoring DNA-damage responses. Noteworthy, plastid transcription is regulated at multiple levels. Although plant sigma factors belong to the bacterial σ70 family, and thus are generally classified as initiation factors, their specific functions in chloroplast transcription have not been completely elucidated yet [4]. For instance, in bacteria, σ^70^ has been also shown to remain associated with, and modulate the behavior of RNAP during elongation [92]. Besides, expression of plastid genes is highly regulated at post-transcriptional levels, including RNA processing, intron splicing, RNA editing, RNA turnover, and translational control. Each of these processes involves RNA-binding proteins of the pentatricopeptide repeat (PPR) family, which are encoded in the nucleus and targeted to the plastids [93–96]. For example, the Arabidopsis *delayed greening1 (dg1)* and *yellow seedling1 (ys1)* mutants display albino and yellow seedling phenotypes, respectively. DG1 was proposed to be involved in the regulation of PEP-dependent transcript accumulation, and YS1 was shown to be required for editing of rpoB transcripts [97,98]. Interestingly, despite the mechanism is still elusive, DG1 was demonstrated to functionally interact with SIG6 [99], indicating a possible cross-connection between transcription and post-transcriptional events. Therefore, the regulation of all these transcriptional mechanisms might be of particular importance under conditions of transcriptional burst such as stress responses, during which the coordination of the transcription and replication machineries could be crucial in the management of DNA-damaging transcription-replication conflicts.

## Supporting information

S1 Table Primer sequences. (DOCX)

S1 Fig Graphical representation of the position of the primers used for the detection of rearrangements by semi-quantitative PCR in the plastid genome of *Arabidopsis thaliana*. The image shows the position of the pairs of primers used to detect DNA rearrangements by PCR described in S1 Table. Inward and outward facing primer pairs enable to detect deletions and duplications, respectively.
(PDF)

S2 Fig Accumulation of ptDNA rearrangements in the six Arabidopsis sigma factors mutants. **(A)** Semi-quantitative PCR reactions carried out on total leaf DNA of wild-type (WT), *sig1*, *sig2*, *sig3*, *sig4*, *sig5*, *sig6* and *why1why3* plants grown 14 days on solid media basal (MS) or containing 0.5 μM ciprofloxacin (CIP). Experiments were performed three times performed with duplicate samples (using two independent T-DNA lines for each sigma factor when available). Low cycle amplification of the atpB plastid gene was used as a loading control. Primers pairs used for PCR reactions are indicated on the left side of the gels. Arrows and numbers on the right side of the gels represent the position and size of the DNA ladder bands in kilobases. **(B)** The bar graph represents fold change intensity of ptDNA rearrangements (mean ± standard error) compared to the WT grown on MS, estimated by quantification of the intensity of all the PCR bands shown in A for each primer pair (n = 8). *P*-values are calculated using a two-tailed paired t-test by comparing mutant lines to the WT (ns: not-significant, *: p<0.05).
(PDF)

S3 Fig Loss of SIG6 decreases plastid transcription rates. **(A)** Run-on transcription assays of chloroplast genes in wild type (WT) and *why1why3*, *sig6-1*, and *why1why3sig6-1* mutant plants. Chloroplasts were isolated from rosette leaves 10 days after germination (DAG), counted using a hemocytometer and normalized among the lines. The [^32^P]-labeled transcripts were isolated and hybridized to ~500 bp plastid gene probes blotted on a Nylon membrane. RNAse A treatment was performed as negative control. Experiments were performed in triplicate, and one representative experiment is presented. **(B)** Histogram showing the average radioactive signal intensity ± standard error (n = 3) of each DNA probe. Chloroplast genes psbA, rbcL, psaA, rrn16S and accD are represented in light blue, green, grey, white and orange, respectively. One-way ANOVA, Tuckey t-test (ns: not-significant, ***: p<0.0001).
(PDF)

S4 Fig Plastid DNA coverage for Arabidopsis WT and mutant lines. **(A)** Relative ptDNA levels (mean ± standard error) measured at three sites of the genome by qPCR in WT, *sig6-1*, *why1why3* and *why1why3sig6-1* plants grown for 14 days on soil, normalized to the nuclear genome. Experiments were performed in duplicate. Kruskal-Wallis and Dunns test (ns: not-significant). **(B-D)** Plastid DNA sequencing coverage curves for Arabidopsis WT and mutant lines indicated. Positions were rounded down to 1 kb. All reads mapping to the plastid large inverted repeats (IRs) were only assigned to the first IR. Y axis represents the number of reads per 1,000,000 total plastid reads. The plastid large-single copy region (LSC), the first IR, and the small-single copy region (SSC) are depicted as a long blue bar, a red bar and a short blue bar, respectively.
(PDF)

S5 Fig Plastid rearrangements accumulate in intergenic spacers, non-coding sequences next to tRNAs and some introns in Arabidopsis wild type and mutant lines. **(A-C)** Plastid rearrangements per 100 nucleotide (nt) window overlapping by 50 nt along the plastid genome, normalized to the coverage for 1 million reads, in **(A)** wild type (WT) and sig6-1, **(B)** *why1why3* and *why1why3sig6-1*, **(C)** WT and WT RNAseH1 lines treated with Estradiol (EST) and (D) *why1why3* and *why1why3* RNAseH1 EST-treated lines. All rearrangements mapping to the plastid large inverted repeats (IRs) were only assigned to the first IR. Peaks accumulating higher levels of rearrangements are indicated. A graphical representation of plastid genome of *Arabidopsis thaliana* from 1 to 128,214 nt (without the second IR) is shown above each graph. A zoom-in region spanning the rRNA operon is represented to the right.
(PDF)

S6 Fig Modulation NEP-dependent transcription affects plastid genome stability. **(A)** PCR analysis of ptDNA rearrangements in 14-d-old wild type (WT) and *rpotp* plants on MS or with 0.5 μM CIP. **(B)** The bar graph represents the fold change intensity (mean ± standard error) of the PCR bands in A respect to WT on MS for each primer pair (n = 5). All PCR experiments were performed at least three times with duplicate samples. Low cycle amplification of the atpB plastid gene was used as a loading control. Primers pairs used for PCR reactions are indicated on the left side of the gels. Arrows and numbers on the right side of the gels represent the position and size of the DNA ladder bands in kilobases. *P*-values were generated by a two-tailed paired t-test, (ns: not-significant, **: p<0.01). **(C)** Representative photographs of the plants described in **A.**
(PDF)

S7 Fig Arabisopsis transgenic lines expressing RNAse H1. **(A)** Diagram of the expression of RNAse H1 induced with β-estradiol. Expression of the inducible cassette is driven by promoter UBQ10. Sequence of the XVE fusion protein is followed by a 3’UTR, pea rbcS E9, and LexA operator sequence. The minimal 35S promoter drives the expression of *E*. *coli* RNAse H1 protein fused to rbcS1 target peptide in N-terminal and StrepII tag in C-terminal. The transcription is stopped by the Nos-terminator. **(B)** Relative RNAse H1 expression levels (mean ± standard error) measured by qRT-PCR in control (Ctl_A and B, wild type plants transformed with the empty vector) and RNAse H1_A, B and C transgenic lines grown for 14 days on solid basal media alone (MS) or with 50 μM β-estradiol (EST), normalized to the expression of the nuclear gene *β-Tubulin*. The values were acquired from three independent experiments, and the expression level of RNAse H1_A EST-induced was adjusted to 1. One-way ANOVA and Tukey t-test (ns: not-significant, ***: p<0.001). **(C)** Representative Western blot performed on total Ctl_A/B and RNAse H1_A chloroplast proteins obtained from seedlings grown on soil and spray-induced with 100 μM EST for 14 days. Antibody against StrepII was used to visualize expression of RNAse H1 and antibody against RBCL was used as a loading control. **(D)** Dot-blot showing the effect of exogenous RNAse H1 expression on the accumulation of plastid R-loops. Plastid DNA was extracted from control (Ctl_A, RNAse H1_A, *why1why3*, and *why1why3* RNAse H1_A lines grown for 14 days on soil, and serial dilutions were spotted and probed with the S9.6 antibody. Experiments were carried out on biological triplicates. **(E)** Histograms showing the fold change intensity (mean ± standard error) of the dot-blot assays described in D (n = 3). Two-tailed paired t-test, (ns: not-significant, * p<0.05). **(F)** Representative PCR analysis carried out on total DNA from control (Ctl_A), *why1why3*, RNAse H1_A, B, C and *why1why3* RNAse H1_A lines grown 14 days on solid media supplemented with 0.5 μM ciprofloxacin alone (CIP, non-induced) or with 50 μM β-estradiol (CIP+EST, induced). PCR results shown are representative of at least three independent experiments. Low cycle amplification of the atpB plastid gene was used as a loading control. Primer pairs used for each PCR reaction are shown next to the gels. Arrows and numbers on the right side of the gels represent the position and size of the DNA ladder bands in kilobases. **(G)** Representative photograph of 14-d-old plants used in F grown on CIP+EST. **(H)** The bar graphs represent the fold change intensity (mean + standard error) of the PCR bands in F for each plant line with respect to Ctl_A grown on CIP without EST treatment. Two-tailed paired t-test, (ns: not-significant, *: p<0.05).
(PDF)

S8 Fig RNAse H1 expression reduces the accumulation of ptDNA rearrangements in *why1why3*. **(A)** Representative PCR analysis carried out on total DNA from control (Ctl_A and B), RNAse H1_A, B, C, *why1why3*, and *why1why3* RNAse H1_A lines grown 14 days on solid media supplemented with 50 μM β-estradiol alone (EST) or in combination with 0.5 μM ciprofloxacin (EST+CIP). PCR results shown are representative of at least three independent experiments. Low cycle amplification of the atpB plastid gene was used as a loading control. Primer pairs used for each PCR reaction are shown next to the gels. Arrows and numbers on the right side of the gels represent the position and size of the DNA ladder bands in kilobases. **(B)** The bar graphs represent the fold change intensity (mean + standard error) of the PCR bands in **A** for each plant line with respect to Ctl_A grown on EST. Two-tailed paired t-test, (ns: not-significant, *: p<0.05).
(PDF)
