# Supplementary material for: Transcription is a major driving force for plastid genome instability in Arabidopsis
Source: PLoS One. 2019 Apr 3;14(4):e0214552. doi: 10.1371/journal.pone.0214552 (PMC6447228; doi:10.1371/journal.pone.0214552)
Supplement: S1 Table — (DOCX) [file pone.0214552.s001.docx]

**S1 Table. Primer sequences.**

| **Primer paires used for detecting plastid DNA rearrangements** | | | |
| --- | --- | --- | --- |
| **Name** | **Sequence (5'-3')** | **Product (bp)** | **Pair name** |
| 50251 F | GGCCCCTTCCTAATTGGAGAT | 10122 | 2 |
| 40130 R | GGGAAGAGGAGGAACATGTCA |  |  |
| 95260 F | AACCTGCTAGTGGAGGAAGAC | 10500 | 3 |
| 84761 R | CCTTAGGCACGGCCATACATA |  |  |
| 116007 F | GCGTTACTCAAGCCGACATTC | 10047 | 4 |
| 105961 R | AAACCACTCATGCCCGGCAAT |  |  |
| 59291 F | AGGCCTAGTCTTTCCGGGAAT | 10343 | 5 |
| 69633 R | GAGGAACATGTACTCGGGTGT |  |  |
| 121141 F | AACGTCATCCGGTTACCGGTT | 9812 | 6 |
| 111330 R | TCAACAGCGGGATTAACCGCA |  |  |
| 59311 F | AGGCCTAGTCTTTCCGGCAAT | 8799 | 7 |
| 50513 R | GAGACGTCTACCACAACAACC |  |  |
| 79920 F | CCATGCCGTTCGGAAGTGA | 11100 | 8 |
| 68821 R | ACGCGGATATATAGCTCGGAG |  |  |
| 30511 F | GCTGCCTCCTTGAAAGAGAGA | 20282 | 9 |
| 10230 R | TGGCAAGAGGTCAACGATTGC |  |  |
| PSBD - 31900 F | GCGTTCCTGCCTAGAGAAAGAGAG | 14570 | psbD - 1 |
| PSBD - 17341 R | GATTTCGCCCAAGTCTGGCATTCTTC |  |  |
| PSBD - 62821 F | GGGGTGTGATGTAACTCTGTCGTTATTG | 30149 | psbD - 2 |
| PSBD - 32700 R | CCAAGGGCTATAGTCATAGCGATCCTC |  |  |
| 498 F | CCATGTACGAGGATCCCCAC | 5024 | 347 rearrangements |
| 499 R | CGGCAATCCTAGGGTTGCTC |  |  |
| ATPB F | ACCTGGGACGTATCGCCC | 565 | loading control 1 |
| ATPB R | CGAGTCCGTTCGCCTACTCC |  |  |
| YCF2 F | GATCTCTGAGAGCTGTTTCCG | 299 | loading control 2 |
| YCF2 R | TGTTTCGCCTCTTACTCGGAG |  |  |
|  | | | |
| **Primer pairs used for qPCR DNA assays** | | | |
| **Name** | **Sequence (5'-3')** | **Product (bp)** | **Pair name** |
| 121141 F | AACGTCATCCGGTTACCGGTT | 9812 | 6 |
| 111330 R | TCAACAGCGGGATTAACCGCA |  |  |
| 59311 F | AGGCCTAGTCTTTCCGGCAAT | 8799 | 7 |
| 50513 R | GAGACGTCTACCACAACAACC |  |  |
| YCF2 F | GATCTCTGAGAGCTGTTTCCG | 299 | ycf2 (housekeeping control) |
| YCF2 R | TGTTTCGCCTCTTACTCGGAG |  |  |
| psbA-CREII F_1 | CCAAGCCGCTAAGAAGAAAT | 167 | psbA at CREII cleavage site |
| psbA-CREII R_1 | TGATCAGGGAAACCACAGAA |  |  |
| psbA-CREII F_2 | TGTATTCCAGGCTGAGCACA | 217 | psbA at CREII cleavage site |
| psbA-CREII R_2 | GGCCAAAATAACCGTGAGCA |  |  |
| 45345 F | TTGGCAATTCCTCAGGGGCAG | 180 | LSC middle (housekeeping control) |
| 45525 R | TTGACTATTCCTCAAGCGCGCC |  |  |
|  | | | |
| **Primer pairs used for RT-PCR and qRT-PCR assays** | | | |
| **Name** | **Sequence (5'-3')** | **Product (bp)** | **Pair name** |
| RNAseH F | TCTTTGGCAGCGTCTTGATG | 130 | RNAse1H1 |
| RNAseH R | AGTTGGATTCATCGCAGCAG |  |  |
| UBQ5 F | GTAAACGTAGGTGAGTCCA | 256 | UBQ5 |
| UBQ5 R | GACGCTTCATCTCGTCC |  |  |
| β-Tubulin F | TCGTTGGGAGGAGGCACAGGT | 243 | TUB5 |
| β-Tubulin R | GCTGAG TTTGAGGGTACGGAAGCAG |  |  |
| 7660 F | TGATCCAGGAC GTAATCCGGGAC | 142 | LSC begining |
| 7802 R | CGAATCCCTCTCTTTCCCCTTCTCC |  |  |
| 45345 F | TTGGCAATTCCTCAGGGGCAG | 180 | LSC middle |
| 45525 R | TTGACTATTCCTCAAGCGCGCC |  |  |
| 81312 F | AGCTACCCAATACTCAGGGGATCC | 148 | LSC end |
| 81460 R | AAATAGAAGCAGGGCGACGCG |  |  |
|  | | | |
| **Primer paires used for run-on transcription assays** | | | |
| **Name** | **Sequence (5'-3')** | **Product (bp)** | **Pair name** |
| PASBA F | GCGAAAGCCTATGGGGTCGC | 600 | psbA |
| PSBA R | GGGAGCCGCCGAATACACC |  |  |
| RBCL F | GAAGCAGGGGCTGCGGTA | 590 | rbcL |
| RBCL R | TCGCATGTACCCGCAGTAGC |  |  |
| PSAA F | CGTTCGCCGGAACCAGAAGT | 624 | psaA |
| PSAAR | TGCCCAAGAAAGGGACCCAAG |  |  |
| RRN16S F | ATGAACGCTGGCGGCATG | 618 | rrn16S |
| RRN16S R | GCATTTCACCGCTCCACCG |  |  |
| ACCD F | GGTGACAACGATCTGCACTATGATCC | 585 | accD |
| ACCD R | CATAACCCCAAGGGCTACCGG |  |  |
|  | | | |
| **Primer pairs used for RNAseH sequencing** | | | |
| **Name** | **Sequence (5'-3')** | | |
| LexA-XVE F | CCGGTCTTGCATCCAGCTGGGC | | |
| LexA-XVE R | GGCTAGAGTCGACTAGCTTC | | |
| XVE-Ctag-MCS | TCTAGAGTTAACCGGGCTCAGGC | | |
| XVE-NOS | GATAATCATCGCAAGACCGGCAACAG | | |
|  | | | |
| **Primer pairs used for identification of mutant and transgenic lines** | | | |
| **Name** | **Sequence (5'-3')** | | **Pair name** |
| LBb1.3 | ATTTTGCCGATTTCGGAAC | | left border for Salk lines |
| SAIL LB3 | TAGCATCTGAATTTCATAACCAATCTC | | left border for Sail lines |
| sig1-1 F | CCTGTTCAGGGATTTCAGCTCG | | *sig1-1* |
| sig1-1 R | CGCAAAAGTCCGATAAGACCACC | |  |
| sig1-1 F + LBb1.3 | | |  |
| sig1-2 F | GGAGAACAATCCGTGGCACG | | *sig1-2* |
| sig1-2 R | GCGGAGTATTATCGCTTTCGCA | |  |
| sig1-2 F + LBb1.3 | | |  |
| sig2-1 F | CCGAACCTCTTTCTGTGCCC | | *sig2-1* |
| sig2-1 R | GAAGGACCATCGGGCCTTG | |  |
| sig2-1 R + LBb1.3 | | |  |
| sig2-2 F | GAGCTGTCGGCAGGAATACAG | | *sig2-2* |
| sig2-2 R | AAAGGTTGGCTGACGTCCAC | |  |
| sig2-2 F + LBb1.3 | | |  |
| sig3-2 F | GCGTCATCGGGCTACCAG | | *sig3-2* |
| sig3-2 R | GCAACGAAACGGAGAGAGGG | |  |
| sig3-2 F + LBb1.3 | | |  |
| sig3-4 F | GGTGGTCTTCTCACTCTAGGGAC | | *sig3-4* |
| sig3-4 R | CTGGTAGCCCGATGACGC | |  |
| sig3-4 F + LBb1.3 | | |  |
| sig4-1 F | GGTGAGTCGTTCTGAGTAAGGGAG | | *sig4-1* |
| sig4-1 R | CGTGGGGTCTAGACCGTGA | |  |
| sig4-1 F + LBb1.3 | | |  |
| sig4-3 F | CCTCTTCTTCAGGCTCCAAACG | | *sig4-3* |
| sig4-3 R | CATCCATGGCGACGACGATTC | |  |
| sig4-3 F + LBb1.3 | | |  |
| sig5-1 F | GTTGAGTGAGGCGGCTCAG | | *sig5-1* |
| sig5-1 R | GCTCGCTCTCGACGATGTG | |  |
| sig5-1 R + LBb1.3 | | |  |
| sig5-2 F | GCTGAATAGTCCCGCACAAGC | | *sig5-2* |
| sig5-2 R | CGTTGCCCAACCCGAGTG | |  |
| sig5-2 R + LBb1.3 | | |  |
| sig6-1/2 F | CGTCTCTCTAGCTGCCGCTTCG | | *sig6-1/2* |
| sig6-1/2 R | CGGGAAGAGTCTAGGCCTCTG | |  |
| sig6-1/2 R + SAIL LB3 | | |  |
| why1 F | GGCCAATCGTTGGTCCTAAAATCG | | *why1* |
| why1 R | TGACCCACGTAAAATCTAGCAGGC | |  |
| why1 F + LBb1.3 | | |  |
| why3 F | AAAGGAAGGTTTTTTGTTGCTCCAATTTG | | *why3* |
| why3 R | GCCCAAGGCTAACTAGATTACCGAT | |  |
| PCR why3 F-R + MvaI restriction (if wild type, the product loses ~20 bp) | | |  |
| recA1 F | TCCTCGACCGCGTAAGTTCTT | | *recA1* |
| recA1 R | ACCAGCACTCCCCAATCTTGT | |  |
| recA1 F + LBb1.3 | | |  |
| polIB F | AGAGCAATGGCTACCAGCTCA | | *polIB* |
| polIB R | CGAATATCCCGCATTTCCCT | |  |
| polIB F + LBb1.3 | | |  |
| hsp21 F | ATGGCTTCTACACTCTCATTTGCTGC | | *hsp21* |
| hsp21 R-HindIII | TGTCCATAGTATCTAACATTTGTCGCATCGTCCTCATTGGTGACAAAGGATCCAACA | |  |
| PCR hsp21 F-R + HindIII restriction (if mutant, the product loses ~60 bp) | | |  |
| 347 F (488 F) | AGCGCTATGACTACAAAAAAAACAATACAAT | | 347 genotype |
| 347 R (489 R) | CCTAGGAAACGTTATCCAGCCATATGCAG | |  |
| RNAseH-Chloro F | GCGAACTAGTATGGCATCATCTATGTTGT | | RNAseH genotype |
| RNAseH-Chloro R | GGATCTCGAGAACCTCCACCTGGTA | |  |
