# Supplementary material for: Transcription is a major driving force for plastid genome instability in Arabidopsis
Source: PLoS One. 2019 Apr 3;14(4):e0214552. doi: 10.1371/journal.pone.0214552 (PMC6447228; doi:10.1371/journal.pone.0214552)

S1 Fig. Graphical representation of the position of the primers used for the detection of rearrangements by semi-quantitative PCR in the plastid genome of Arabidopsis thaliana.

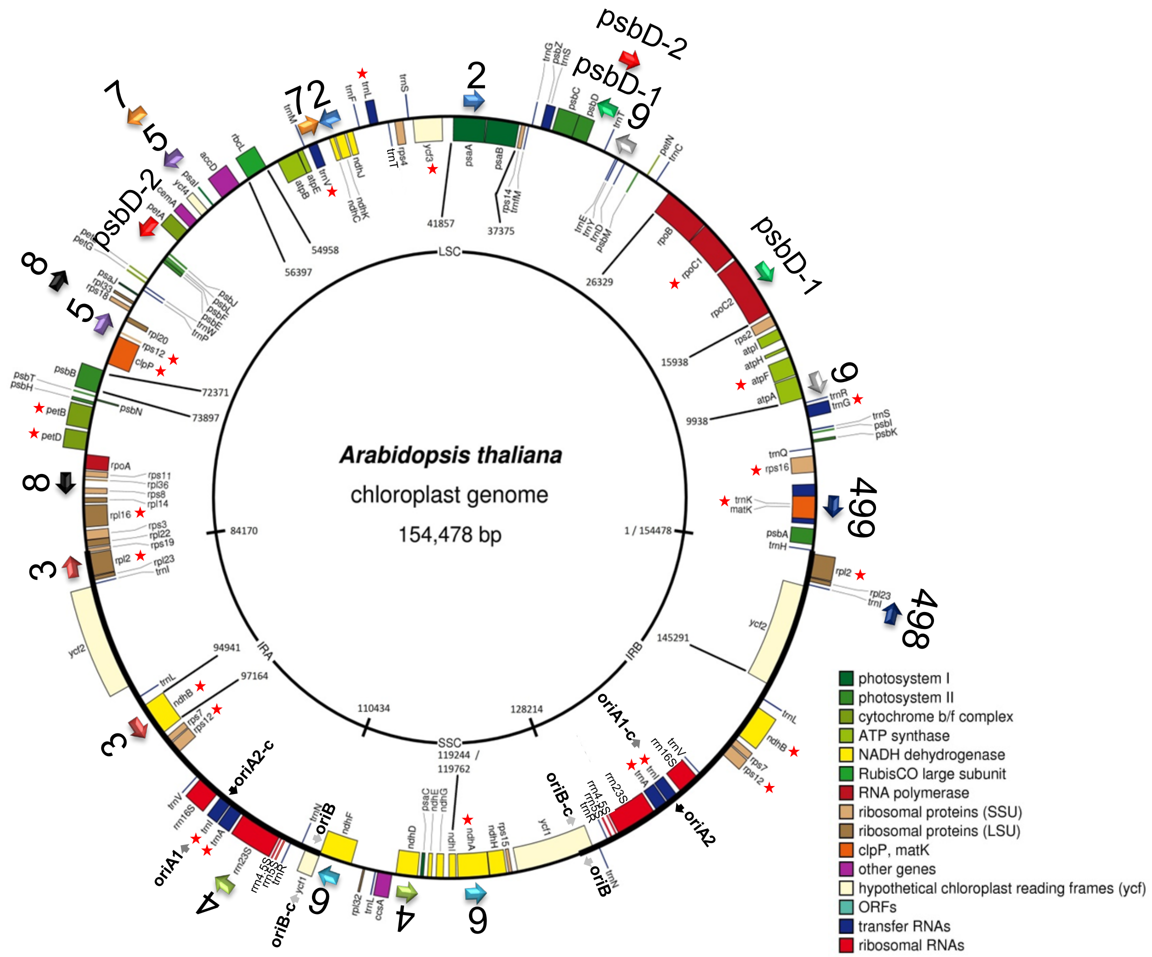

Supplement: S1 Fig — The image shows the position of the pairs of primers used to detect DNA rearrangements by PCR described in S1 Table. Inward and outward facing primer pairs enable to detect deletions and duplications, respectively. (PDF) [file pone.0214552.s002.pdf]
