# Supplementary figures and images for: Transcription is a major driving force for plastid genome instability in Arabidopsis
Source: PLoS One. 2019 Apr 3;14(4):e0214552. doi: 10.1371/journal.pone.0214552 (PMC6447228; doi:10.1371/journal.pone.0214552)

S2 Fig. Accumulation of ptDNA rearrangements in the six Arabidopsis sigma factors mutants.

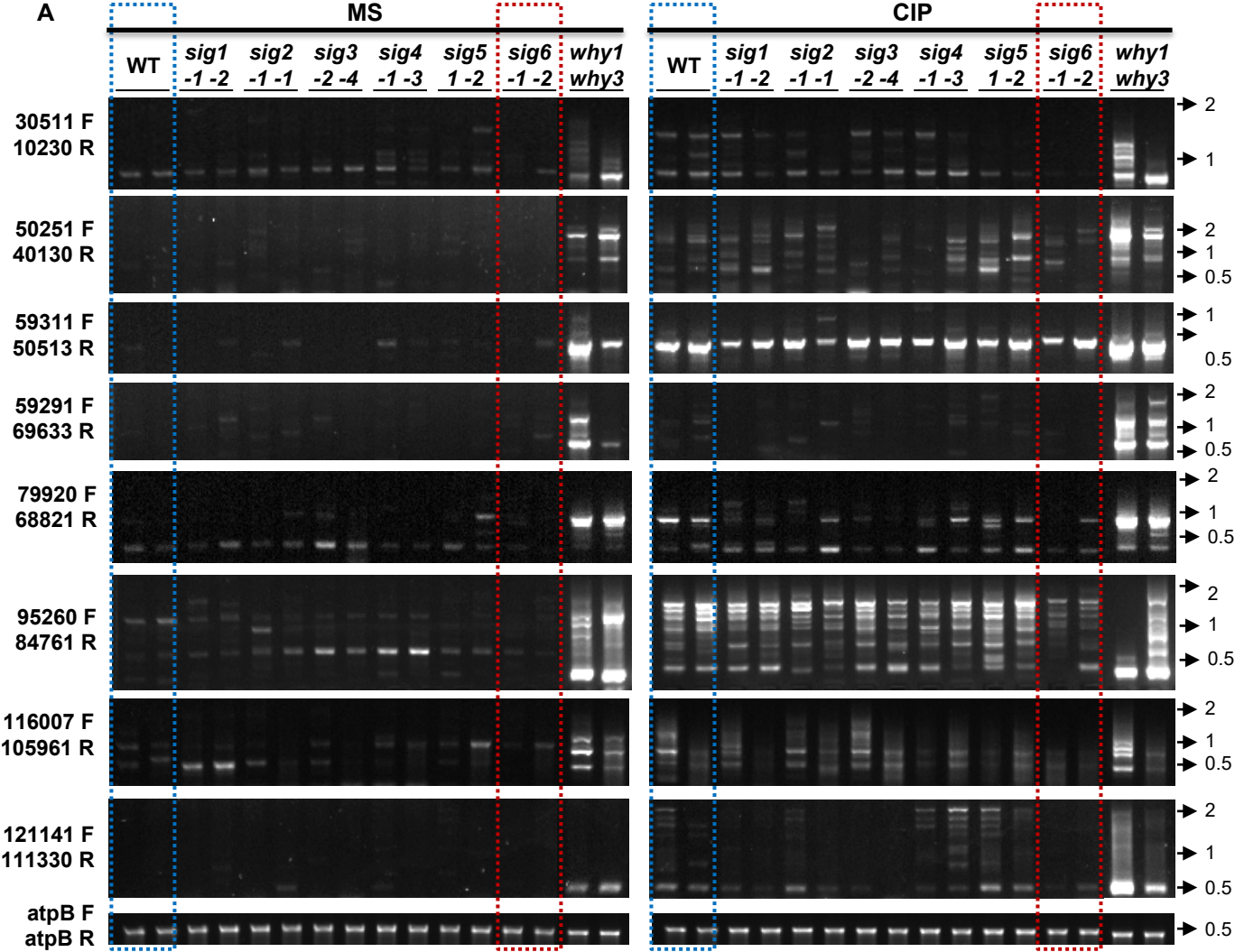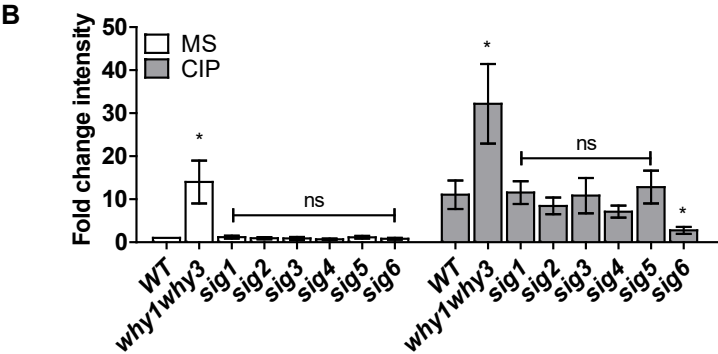

Supplement: S2 Fig — (A) Semi-quantitative PCR reactions carried out on total leaf DNA of wild-type (WT), sig1, sig2, sig3, sig4, sig5, sig6 and why1why3 plants grown 14 days on solid media basal (MS) or containing 0.5 μM ciprofloxacin (CIP). Experiments were performed three times performed with duplicate samples (using two independent T-DNA lines for each sigma factor when available). Low cycle amplification of the atpB plastid gene was used as a loading control. Primers pairs used for PCR reactions are indicated on the left side of the gels. Arrows and numbers on the right side of the gels represent the position and size of the DNA ladder bands in kilobases. (B) The bar graph represents fold change intensity of ptDNA rearrangements (mean ± standard error) compared to the WT grown on MS, estimated by quantification of the intensity of all the PCR bands shown in A for each primer pair (n = 8). P-values are calculated using a two-tailed paired t-test by comparing mutant lines to the WT (ns: not-significant, *: p<0.05). (PDF) [file pone.0214552.s003.pdf]

S3 Fig. Loss of SIG6 decreases plastid transcription rates.

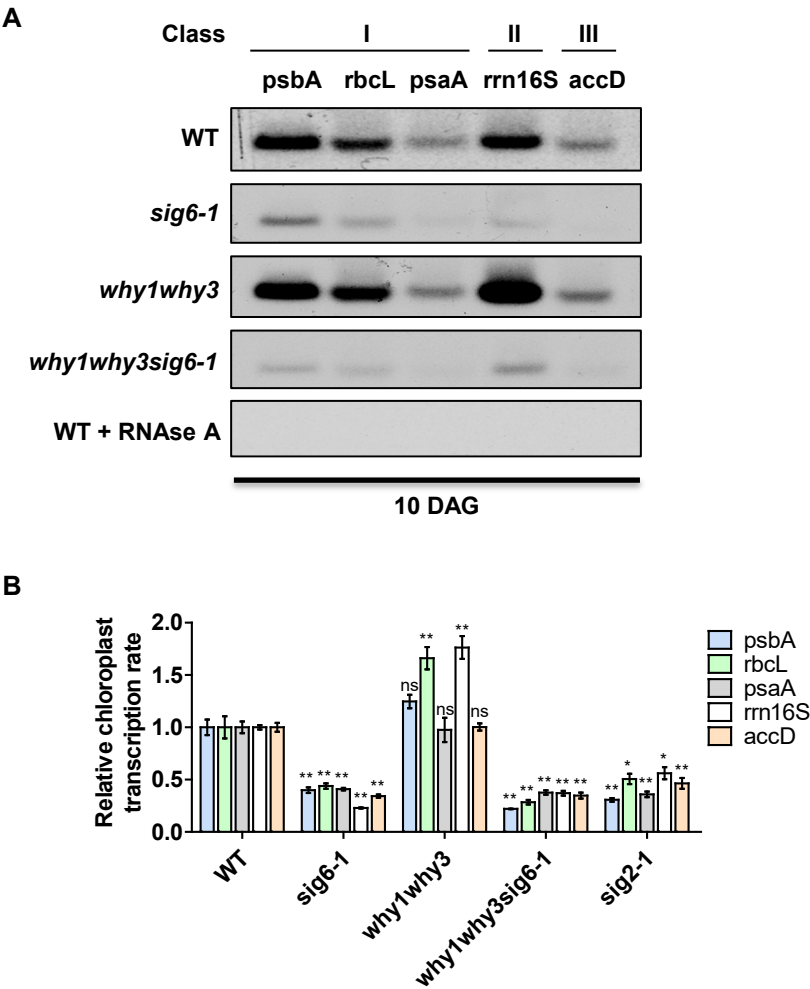

Supplement: S3 Fig — (A) Run-on transcription assays of chloroplast genes in wild type (WT) and why1why3, sig6-1, and why1why3sig6-1 mutant plants. Chloroplasts were isolated from rosette leaves 10 days after germination (DAG), counted using a hemocytometer and normalized among the lines. The [32P]-labeled transcripts were isolated and hybridized to ~500 bp plastid gene probes blotted on a Nylon membrane. RNAse A treatment was performed as negative control. Experiments were performed in triplicate, and one representative experiment is presented. (B) Histogram showing the average radioactive signal intensity ± standard error (n = 3) of each DNA probe. Chloroplast genes psbA, rbcL, psaA, rrn16S and accD are represented in light blue, green, grey, white and orange, respectively. One-way ANOVA, Tuckey t-test (ns: not-significant, ***: p<0.0001). (PDF) [file pone.0214552.s004.pdf]

S4 Fig. Plastid DNA coverage for Arabidopsis WT and mutant lines.

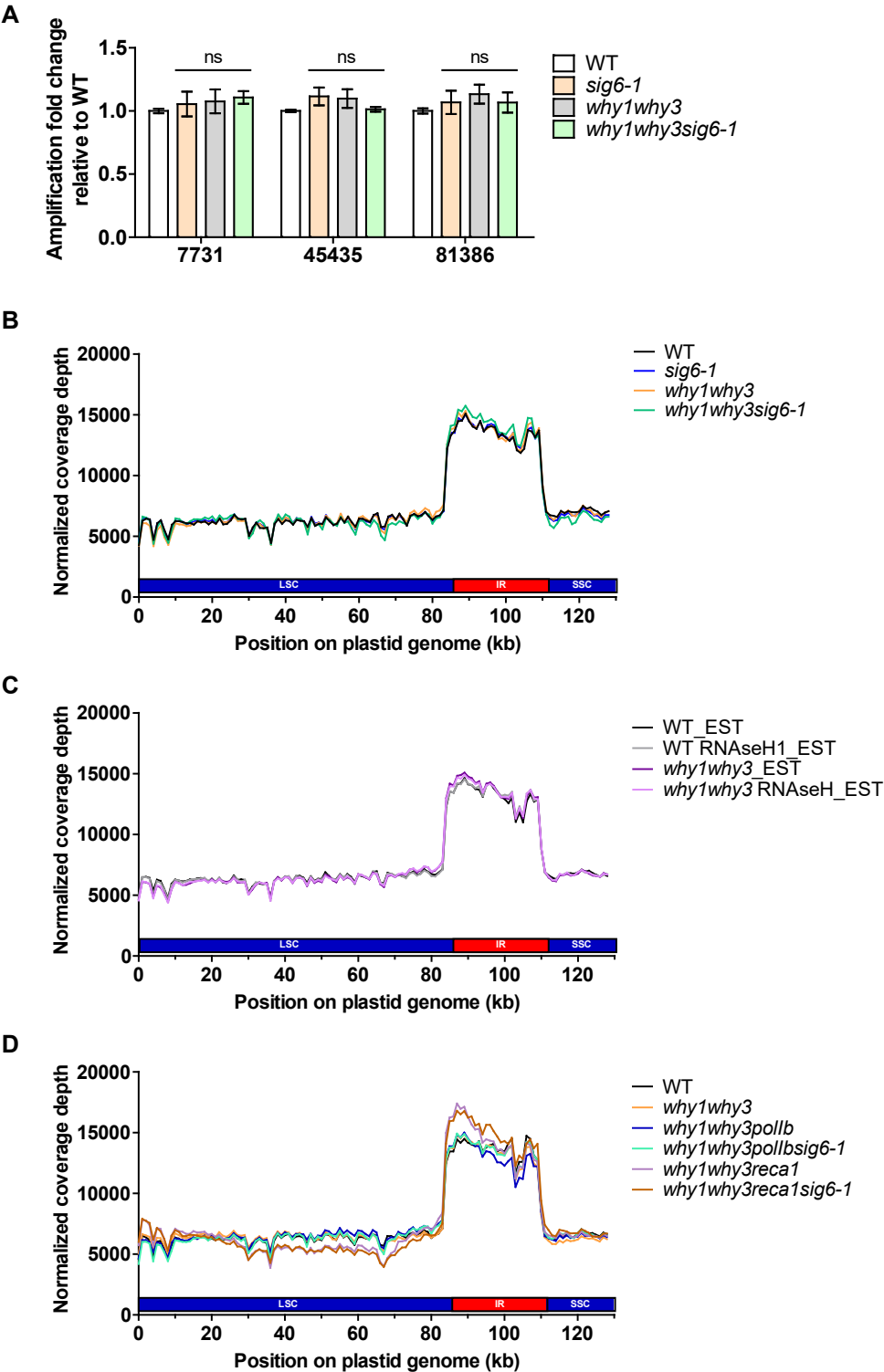

Supplement: S4 Fig — (A) Relative ptDNA levels (mean ± standard error) measured at three sites of the genome by qPCR in WT, sig6-1, why1why3 and why1why3sig6-1 plants grown for 14 days on soil, normalized to the nuclear genome. Experiments were performed in duplicate. Kruskal-Wallis and Dunns test (ns: not-significant). (B-D) Plastid DNA sequencing coverage curves for Arabidopsis WT and mutant lines indicated. Positions were rounded down to 1 kb. All reads mapping to the plastid large inverted repeats (IRs) were only assigned to the first IR. Y axis represents the number of reads per 1,000,000 total plastid reads. The plastid large-single copy region (LSC), the first IR, and the small-single copy region (SSC) are depicted as a long blue bar, a red bar and a short blue bar, respectively. (PDF) [file pone.0214552.s005.pdf]

S6 Fig. Modulation NEP-dependent transcription affects plastid genome stability.

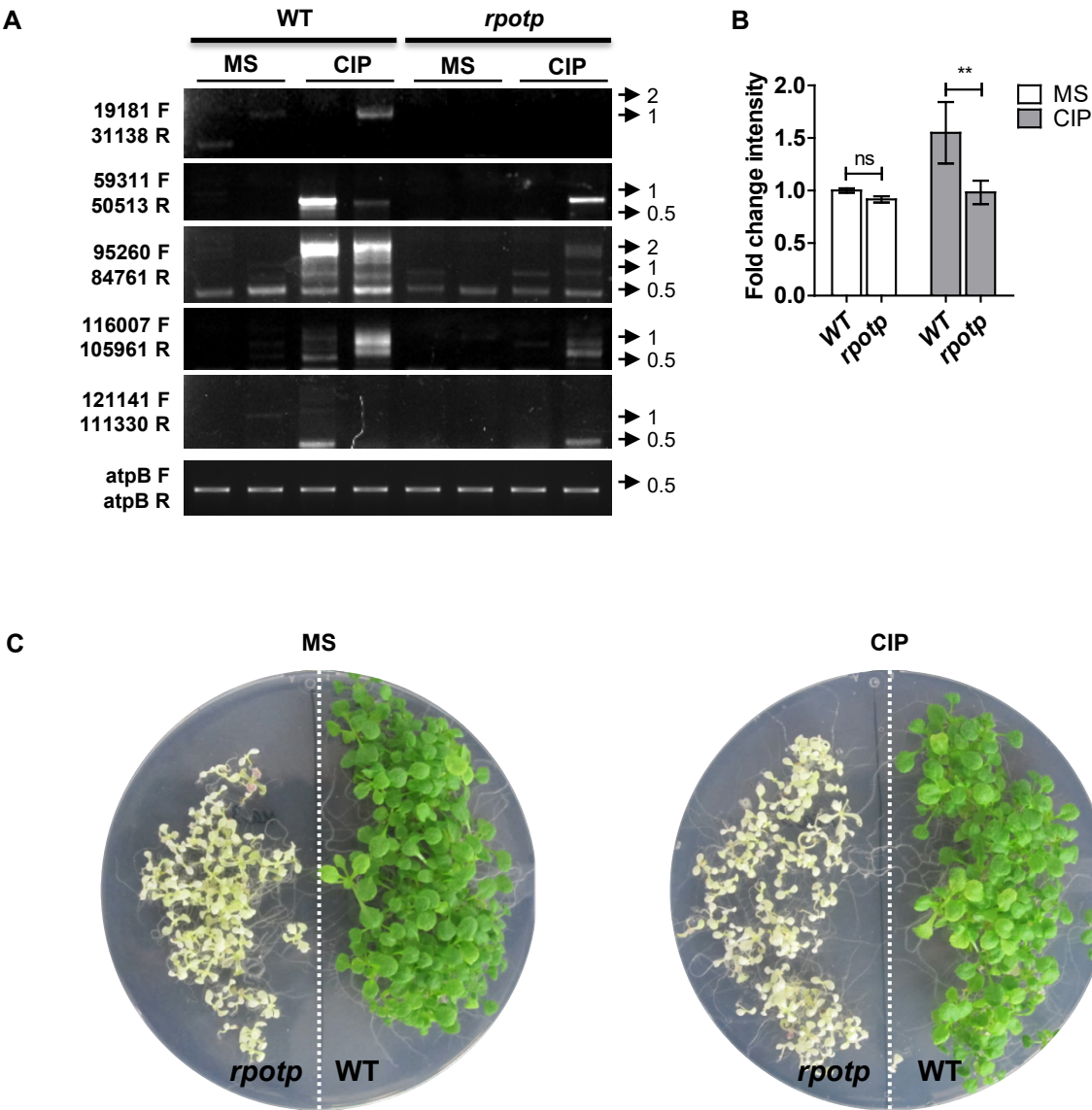

Supplement: S6 Fig — (A) PCR analysis of ptDNA rearrangements in 14-d-old wild type (WT) and rpotp plants on MS or with 0.5 μM CIP. (B) The bar graph represents the fold change intensity (mean ± standard error) of the PCR bands in A respect to WT on MS for each primer pair (n = 5). All PCR experiments were performed at least three times with duplicate samples. Low cycle amplification of the atpB plastid gene was used as a loading control. Primers pairs used for PCR reactions are indicated on the left side of the gels. Arrows and numbers on the right side of the gels represent the position and size of the DNA ladder bands in kilobases. P-values were generated by a two-tailed paired t-test, (ns: not-significant, **: p<0.01). (C) Representative photographs of the plants described in A. (PDF) [file pone.0214552.s007.pdf]

**S7 Fig. Arabisopsis transgenic lines expressing RNase H1.**

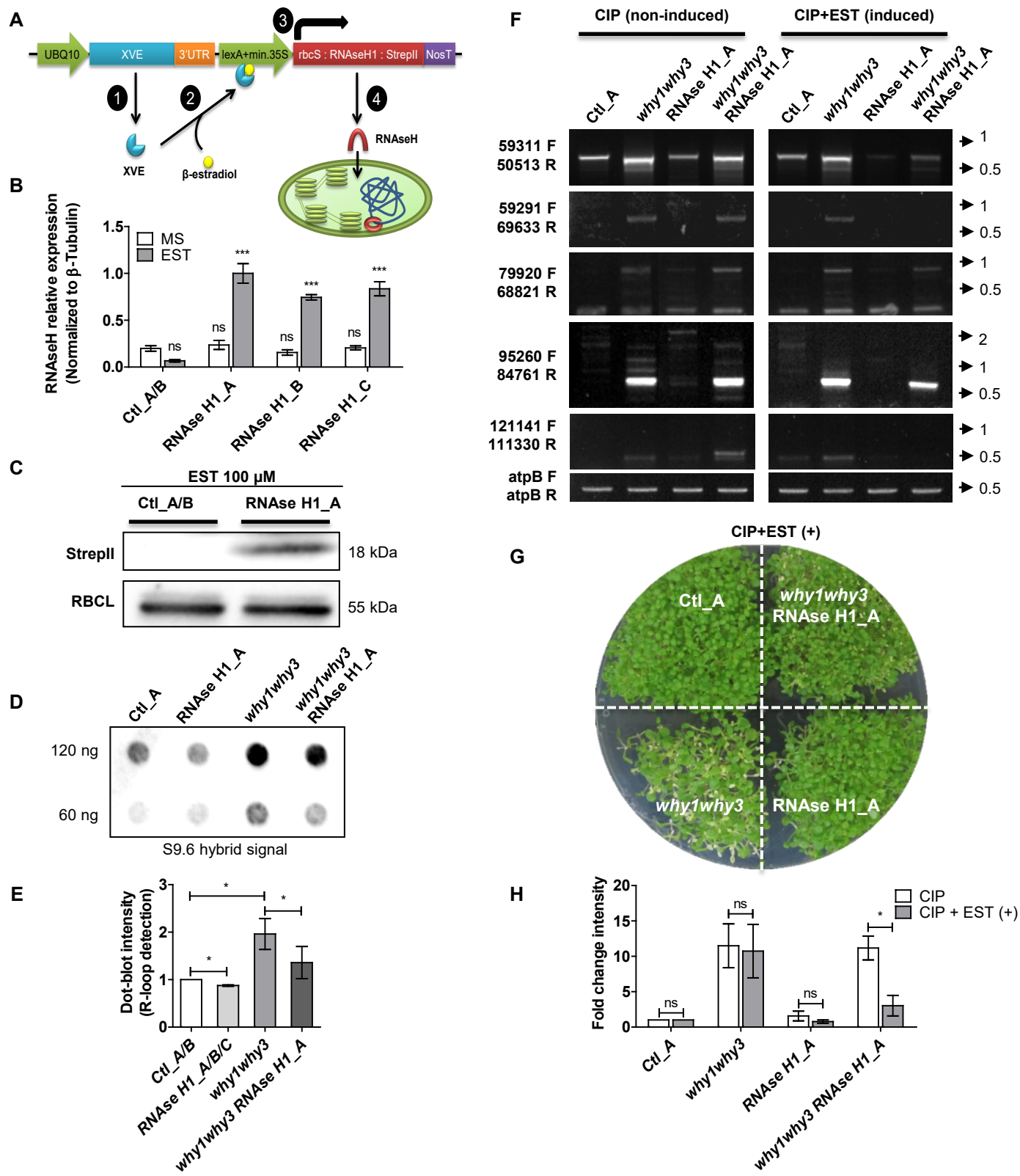

Supplement: S7 Fig — (A) Diagram of the expression of RNAse H1 induced with β-estradiol. Expression of the inducible cassette is driven by promoter UBQ10. Sequence of the XVE fusion protein is followed by a 3’UTR, pea rbcS E9, and LexA operator sequence. The minimal 35S promoter drives the expression of E. coli RNAse H1 protein fused to rbcS1 target peptide in N-terminal and StrepII tag in C-terminal. The transcription is stopped by the Nos-terminator. (B) Relative RNAse H1 expression levels (mean ± standard error) measured by qRT-PCR in control (Ctl_A and B, wild type plants transformed with the empty vector) and RNAse H1_A, B and C transgenic lines grown for 14 days on solid basal media alone (MS) or with 50 μM β-estradiol (EST), normalized to the expression of the nuclear gene β-Tubulin. The values were acquired from three independent experiments, and the expression level of RNAse H1_A EST-induced was adjusted to 1. One-way ANOVA and Tukey t-test (ns: not-significant, ***: p<0.001). (C) Representative Western blot performed on total Ctl_A/B and RNAse H1_A chloroplast proteins obtained from seedlings grown on soil and spray-induced with 100 μM EST for 14 days. Antibody against StrepII was used to visualize expression of RNAse H1 and antibody against RBCL was used as a loading control. (D) Dot-blot showing the effect of exogenous RNAse H1 expression on the accumulation of plastid R-loops. Plastid DNA was extracted from control (Ctl_A, RNAse H1_A, why1why3, and why1why3 RNAse H1_A lines grown for 14 days on soil, and serial dilutions were spotted and probed with the S9.6 antibody. Experiments were carried out on biological triplicates. (E) Histograms showing the fold change intensity (mean ± standard error) of the dot-blot assays described in D (n = 3). Two-tailed paired t-test, (ns: not-significant, * p<0.05). (F) Representative PCR analysis carried out on total DNA from control (Ctl_A), why1why3, RNAse H1_A, B, C and why1why3 RNAse H1_A lines grown 14 days on solid media supplemented [file pone.0214552.s008.pdf]
