# Supplementary material for: Transcription is a major driving force for plastid genome instability in Arabidopsis
Source: PLoS One. 2019 Apr 3;14(4):e0214552. doi: 10.1371/journal.pone.0214552 (PMC6447228; doi:10.1371/journal.pone.0214552)

S5 Fig. Plastid rearrangements accumulate in intergenic spacers, non-coding sequences next to tRNAs and some introns in Arabidopsis wild type and mutant lines.

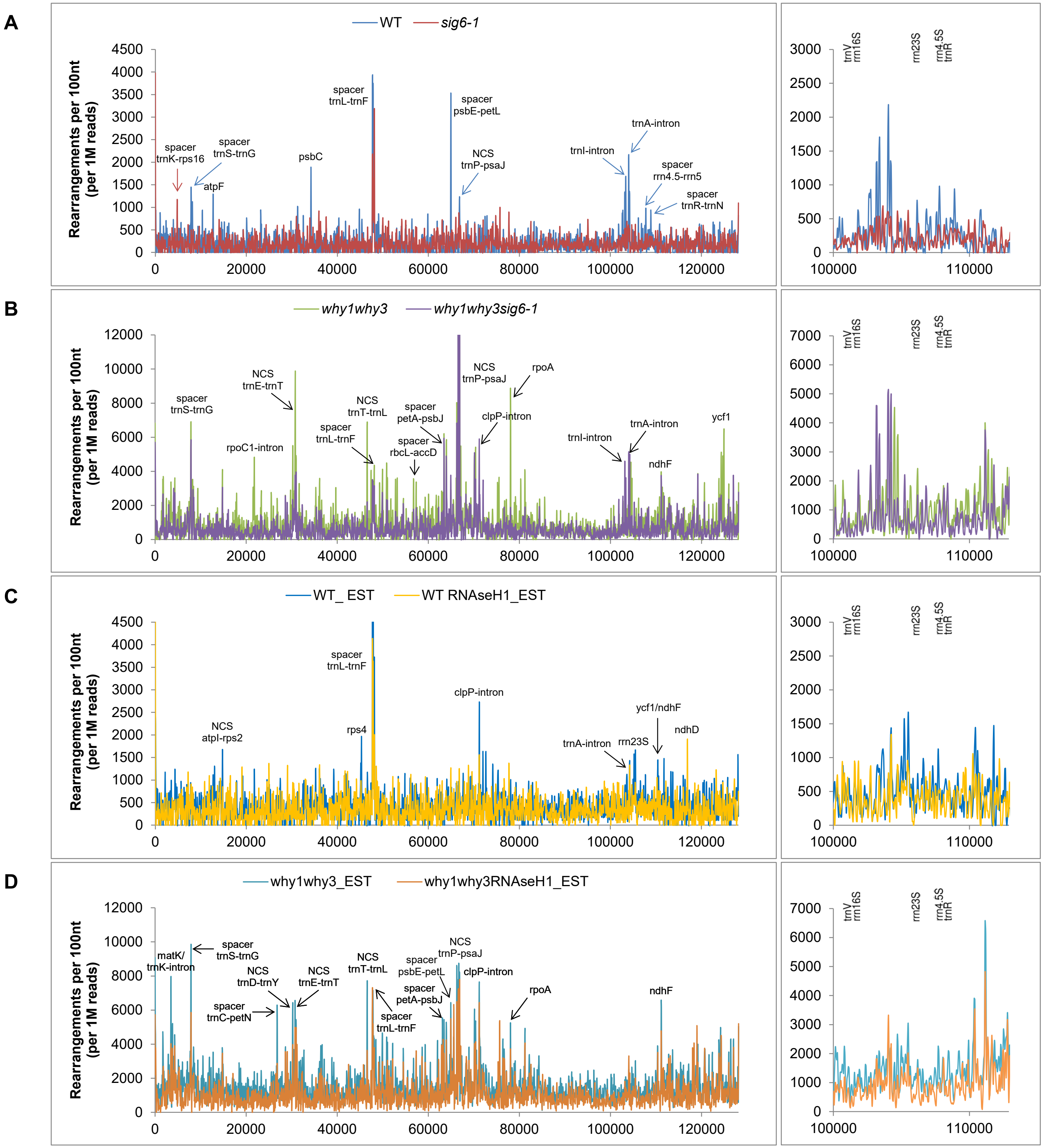

Supplement: S5 Fig — (A-C) Plastid rearrangements per 100 nucleotide (nt) window overlapping by 50 nt along the plastid genome, normalized to the coverage for 1 million reads, in (A) wild type (WT) and sig6-1, (B) why1why3 and why1why3sig6-1, (C) WT and WT RNAseH1 lines treated with Estradiol (EST) and (D) why1why3 and why1why3 RNAseH1 EST-treated lines. All rearrangements mapping to the plastid large inverted repeats (IRs) were only assigned to the first IR. Peaks accumulating higher levels of rearrangements are indicated. A graphical representation of plastid genome of Arabidopsis thaliana from 1 to 128,214 nt (without the second IR) is shown above each graph. A zoom-in region spanning the rRNA operon is represented to the right. (PDF) [file pone.0214552.s006.pdf]
