# Supplementary material for: Transcription is a major driving force for plastid genome instability in Arabidopsis
Source: PLoS One. 2019 Apr 3;14(4):e0214552. doi: 10.1371/journal.pone.0214552 (PMC6447228; doi:10.1371/journal.pone.0214552)

S8 Fig. RNAse H1 expression reduces the accumulation of ptDNA rearrangements in *why1why3*.

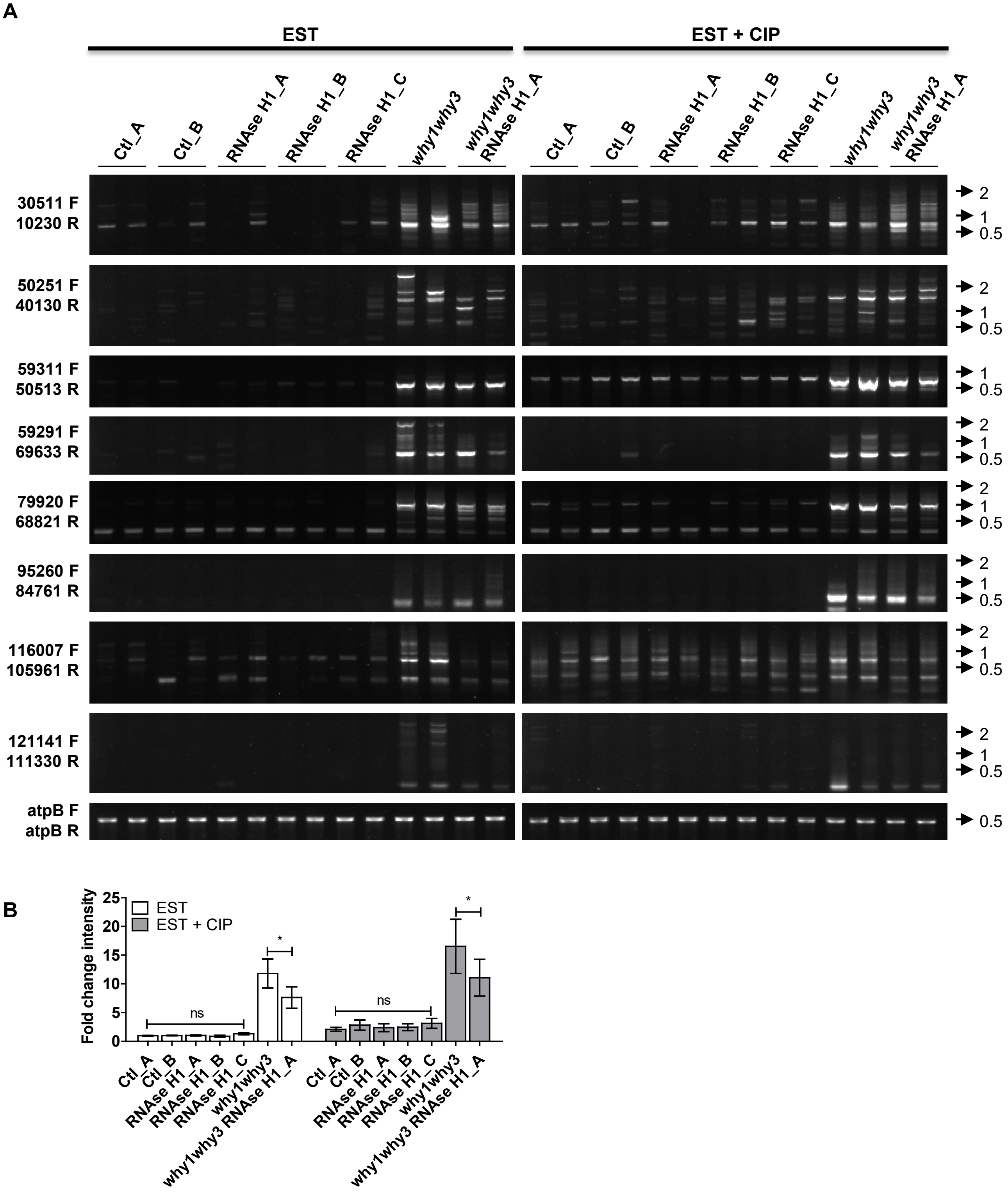

Supplement: S8 Fig — (A) Representative PCR analysis carried out on total DNA from control (Ctl_A and B), RNAse H1_A, B, C, why1why3, and why1why3 RNAse H1_A lines grown 14 days on solid media supplemented with 50 μM β-estradiol alone (EST) or in combination with 0.5 μM ciprofloxacin (EST+CIP). PCR results shown are representative of at least three independent experiments. Low cycle amplification of the atpB plastid gene was used as a loading control. Primer pairs used for each PCR reaction are shown next to the gels. Arrows and numbers on the right side of the gels represent the position and size of the DNA ladder bands in kilobases. (B) The bar graphs represent the fold change intensity (mean + standard error) of the PCR bands in A for each plant line with respect to Ctl_A grown on EST. Two-tailed paired t-test, (ns: not-significant, *: p<0.05). (PDF) [file pone.0214552.s009.pdf]
